# Treatment of mild to moderate community-acquired pneumonia in previously healthy children: an Italian intersociety consensus (SIPPS-SIP-SITIP-FIMP-SIAIP-SIMRI-FIMMG-SIMG)

**DOI:** 10.1186/s13052-024-01786-8

**Published:** 2024-10-19

**Authors:** Daniele Donà, Giulia Brigadoi, Roberto Grandinetti, Laura Pedretti, Giovanni Boscarino, Elisa Barbieri, Luigi Matera, Enrica Mancino, Marcello Bergamini, Guido Castelli Gattinara, Elena Chiappini, Mattia Doria, Luisa Galli, Alfredo Guarino, Andrea Lo Vecchio, Elisabetta Venturini, Gianluigi Marseglia, Maria Carmen Verga, Giuseppe Di Mauro, Nicola Principi, Fabio Midulla, Susanna Esposito

**Affiliations:** 1 Division of Pediatric Infectious Diseases, Department of Women’s and Children’s Health, University of Padua, Padua, Italy; 2 Pediatric Clinic, Department of Medicine and Surgery, Pietro Barilla Children’s Hospital, University Hospital of Parma, Parma, 43126 Italy; 3 Department of Maternal, Infantile and Urological Sciences, Sapienza University of Rome, Rome, Italy; 4 Department of Primary Cares, AUSL Ferrara, Ferrara, 44121 FE Italy; 5 Institute of Child Health, Bambino Gesù Children’s Hospital, IRCCS, Rome, Italy; 6 Department of Health Science, University of Florence, Florence, Italy; 7 Infectious Diseases Unit, Meyer Children’s University Hospital, IRCCS, Florence, Italy; 8 Family Pediatrician, Local Health Unit, Chioggia, Venice, Italy; 9 Department of Translational Medical Sciences, Federico II University, Naples, Italy; 10 Department of Pediatrics, University of Pavia IRCCS San Matteo Foundation, Pavia, Italy; 11 Family Pediatrician, Local Health Unit Salerno, Vietri sul Mare, Salerno, Italy; 12 Pediatric Primary Care, National Pediatric Health Care System, Caserta, Italy; 13 Università degli Studi di Milano, Milan, 20122 Italy; 14 Department of Women’s and Children’s Health, University of Padova, Via Giustiniani 3, Padova, 35141 Italy

**Keywords:** Antibiotic therapy, Community-acquired pneumonia, Pediatric infectious diseases, Primary care, Respiratory infections

## Abstract

Community-acquired pneumonia (CAP) is an acute infection of the lung parenchyma acquired outside the hospital or other healthcare settings, typically affecting previously healthy individuals. This intersociety consensus aims to provide evidence-based recommendations for the antibiotic treatment of mild to moderate CAP in previously healthy children in Italy.

A systematic review was conducted to identify the most recent and relevant evidence. Embase, Scopus, PubMed, and Cochrane databases were systematically screened, with a date restriction from 2012 to April 2024, but without language limitations. The review included studies conducted in high-income countries on antibiotic therapy in children over 3 months of age diagnosed with mild-moderate CAP. The certainty of evidence was assessed using the Grading of Recommendations Assessment, Development, and Evaluation (GRADE) methods. The final recommendations were obtained through a Delphi consensus of an expert panel.

Amoxicillin is the first-line treatment if the child is at least immunized against *Haemophilus influenzae* type b (low/very low quality of evidence, strong recommendations), while amoxicillin-clavulanate or second- or third-generation cephalosporins should be prescribed for those unimmunized or with incomplete immunization coverage for both *H. influenzae* type b and *Streptococcus pneumoniae* (low/very low quality of evidence, strong recommendations). Macrolides should be considered in addition to amoxicillin in children over 5 years old, if symptoms persist and the clinical condition remains good after 48 h of therapy (low/very low quality of evidence, strong recommendations). The dosage of amoxicillin is 90 mg/kg/day divided in three doses, although two doses could be considered to improve compliance (moderate quality of evidence, weak recommendations). A five-day duration of therapy is recommended, with clinical monitoring and re-assessment approximately 72 h after the start of antibiotic treatment to evaluate symptom resolution (moderate quality of evidence, strong recommendations).

To improve the management of CAP in pediatric patients, we have developed this consensus based on a thorough review of the best available evidence and extensive discussions with an expert panel. However, further efforts are needed. Future research should focus on enhancing diagnostic accuracy, optimizing antibiotic utilization, comparing the efficacy of different antibiotic regimens, and determining the optimal dosage and duration of treatment in different setting.

**Supplementary Information:**

The online version contains supplementary material available at 10.1186/s13052-024-01786-8.

## Background

Community-acquired pneumonia (CAP) is an acute infection of the lung parenchyma acquired outside the hospital or other healthcare settings, typically affecting previously healthy individuals.

Although the discovery of penicillin and the introduction of immunization against *Haemophilus influenzae* type b and *Streptococcus pneumoniae* have significantly reduced the incidence and the mortality of pediatric pneumonia [1], this infectious disease remains a common cause of hospitalization and death in children worldwide, especially in low and middle-income countries, accounting for 4% of neonatal and 15% of pediatric deaths annually, totaling 1.3 million deaths globally [2–5].

Pneumonia is diagnosed by fever with respiratory symptoms like tachypnea, cough, and chest pain, often confirmed by clinical or radiological evidence of lung consolidation [6]. Diagnosis is mostly clinical, with chest X-rays reserved for severe cases [7]. Hospitalization for pneumonia depends on disease severity, respiratory failure, and home care limitations [8–10].

CAP can be caused by viruses, bacteria, or co-infections, with causative agents varying by age [9, 11]. In the post-immunization era, viruses account for at least 70% of pediatric CAP cases, with common pathogens including RSV, Rhinovirus, Influenza, Metapneumovirus, and Adenovirus, especially in children under five. Viral pneumonia represents the majority of community-managed CAP cases [7, 12]. Common bacterial pathogens include *S. pneumoniae*, *H. influenzae*, and *Mycoplasma pneumoniae* [8, 13, 14].

Determining the etiology of CAP is crucial for treatment but challenging due to the overlap between the clinical presentation of viral and bacterial infections [6], as well as unreliable diagnostic tests and difficulties in sample collection [1, 15, 16].

Managing a child with suspected CAP is challenging for primary care paediatricians due to a lack of diagnostic tools, clear severity criteria, and reliable laboratory methods to guide antibiotic use. This leads to varying treatment recommendations.

To assess the most recent evidence regarding the treatment of mild to moderate CAP in previously healthy children in high-income countries, focusing on antibiotic choice, dosage, and duration of therapy, we conducted a systematic review of the literature. Our findings, combined with insights gathered through a Delphi consensus among experts, enabled us to develop comprehensive, evidence-based recommendations for clinical practice.

## Methods

This article is one of the four papers of the Italian intersociety consensus on the judicious use of antibiotic therapy in respiratory tract infections in childhood (sinusitis, otitis, pharyngitis and CAP) involving the Italian Society for Preventive and Social Paediatrics (SIPPS), the Italian Society for Paediatrics (SIP), the Italian Society of Pediatric Infectious Disease (SITIP), the Italian Federation of Paediatricians (FIMP), the Italian Society for Paediatric Allergy and Immunology (SIAIP), the Italian Society of Paediatric Respiratory Diseases (SIMRI), the Italian Federation of General Practitioners (FIMMG), the Italian Society of General Medicine and Primary Care (SIMG). The Consensus protocol is published in the supplementary material (Supplement, Sect. 1).

A systematic review was reported following the PRISMA checklist (Preferred Reporting Items for Systematic Reviews and Meta-analyses) [17] to elaborate recommendations regarding the choice of antibiotics, the dosage, and the duration of therapy for children with mild-moderate pneumonia.

Embase, Scopus, PubMed, and Cochrane databases were systematically screened, combining the terms “children,” “community-acquired pneumonia,” and “antibiotics,” with a date restriction from 2012 to April 2024, but without language limitations. The complete search strategy is published in the supplementary material (Supplement, Sect. 2). The review included studies conducted in high-income countries (HICs) on antibiotic therapy in children over 3 months of age diagnosed with mild-moderate CAP. Randomized controlled trials, observational studies, and systematic reviews with or without meta-analysis were included.

Regarding the systematic reviews, a meticulous assessment was conducted to determine if the same article was included in multiple systematic reviews or analyzed as a single study article. In instances where a meta-analysis was performed, the results were taken into account only if the articles included were not already covered in other meta-analyses. If one or more articles from a meta-analysis had already been considered, individual studies not included in other systematic reviews with meta-analyses were examined. For systematic reviews without meta-analyses, individual studies included were evaluated separately. This approach was taken even for studies published before 2012 if they were deemed relevant to the research questions.

Studies conducted in low- and middle-income countries (LMICs) were excluded due to differences in epidemiology, the presence of comorbidities such as HIV and malnutrition that could complicate the typical course of illnesses, and variations in healthcare system structures and resources.

Additionally, studies focusing on patients with complicated pneumonia, whether treated in the community or hospital setting, were excluded. Furthermore, studies that were not relevant to the considered population, intervention, comparison, or outcomes were also excluded from the analysis.

The outcomes considered were:

- Treatment failure, defined as the need for a change in antibiotic therapy within the first 14 days due to persistent symptoms or a new prescription of antibiotics within 30 days for a new episode of pneumonia, hospitalization, progression to severe/complicated pneumonia or intensive care unit admission, persistence of fever at 72 h, or persistence of cough after 5 days from the start of antibiotic therapy.
- Severe adverse events requiring discontinuation of ongoing antibiotic therapy.
- Development of antibiotic resistance following antibiotic therapy.

Two subgroup analyses were conducted, comparing the immunized population (defined as double-dose immunization against *H. influenzae* type b and *S. pneumoniae*) with the unimmunized population and the population aged over or under 5 years, considering that the prevalence of atypical bacteria pneumonia is higher in children older than 5 years of age compared to younger children.

Regarding immunization status, children were considered not immunized if enrolled before 2000. For articles published after 2000, if immunization status was not specified, factors such as the country where the study was conducted, the years when subjects were enrolled, the introduction date of vaccines in that specific country, and possibly the percentage of immunized subjects according to institutional reports were considered [18–25]. If not specifically reported in the article, the population was considered completely immunized if the immunization coverage in the specific countries during the patient enrollment period was > 80%.

Depending on the type of study, the risk of bias was evaluated using the Amstar tool [26] and the Newcastle Quality Assessment Scale [27]. The certainty of evidence was assessed using the Grading of Recommendations Assessment, Development, and Evaluation (GRADE) methods [28]. The final recommendations were obtained through a Delphi consensus of an expert panel (see Supplementary material, Sect. 1).

Many robust studies, particularly randomized clinical trials comparing amoxicillin to other antibiotics, were published prior to 2010 and were not included in our systematic review. However, these studies informed earlier guidelines, which established strong recommendations based on the available evidence. To address the potential risks of issuing weak recommendations from our review—especially in areas where strong evidence existed before our cut-off—we combined the expert panel’s consensus with the certainty of evidence obtained through GRADE. This approach allowed us to assess the strength of recommendations using both factors, enabling strong recommendations even when the quality of evidence was low or very low, and weak recommendations in the presence of moderate-quality evidence.

## Results and recommendations

**Fig. 1:**
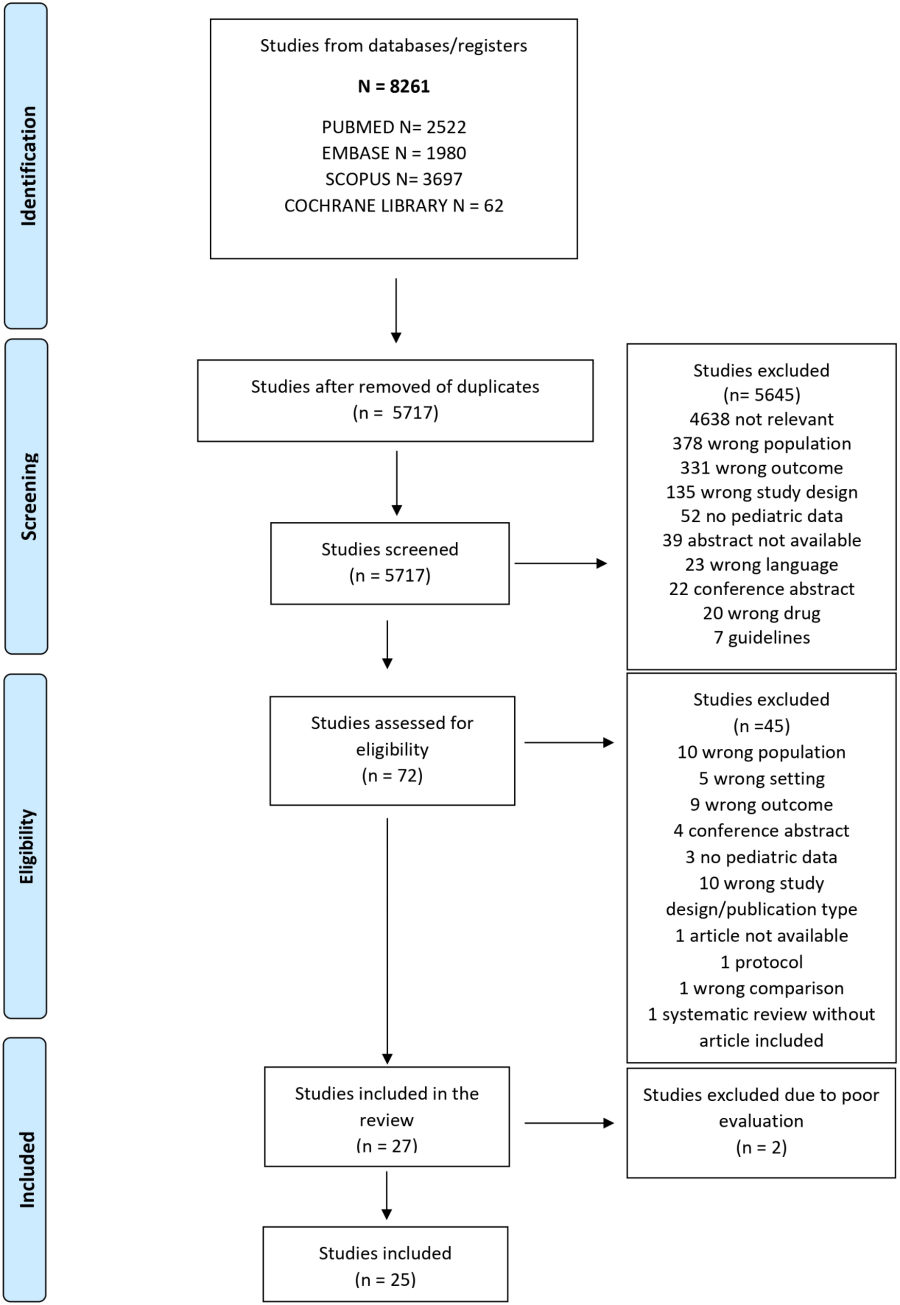
PRISMA flowchart of included studies. Legend: *n*, number

From the systematic search conducted, 8261 articles were identified, and after removing duplicates, 5717 were screened for title and abstract. 77 articles were deemed eligible for review, of which 27 studies were included after a full-text review: 9 systematic reviews [29–37] (including 6 with meta-analyses), 4 randomized controlled trials [38–41], and 14 observational studies [42–55] (including 11 cohort studies and 3 Before and After studies) were finally included (Fig. 1). The list of excluded articles and the reason for exclusion are reported in the supplementary material (Supplement, Sects. 3 and 4). For the review by Lodha et al. [31], which included 29 articles, only the 7 articles conducted in HIC countries were considered available for the current study, whereas those conducted in LMIC were excluded.

The characteristics of the studies included, and the main outcomes are reported in Table 1.

**Table 1:** Characteristics of included studies 1

**1a. Systematic review**							
**Title, author, year of publication**	**Number and type of studies included, population included**	**Aim of the review**	**Main outcomes**	**Results**	**Conclusions**	**Contributing Evidence**	
Treatment of mycoplasma pneumonia: A systematic review Biondi et al., 2014 [29]	4294 children (< 18 years of age) from 17 studies included studies for the qualitative evaluation 723 children included from 8 studies for the meta-analysis 445 children included from 5 randomized trial included in the meta-analysis	The objective was to provide a more comprehensive review of all available published literature on the use of antibiotics in children to treat CA-LRTI secondary to M. pneumoniae.	The primary outcome was clinical improvement or cure at follow-up. Clinical improvement or cure could include resolution of fever; resolution or improvement in symptoms such as cough, congestion, shortness of breath, fatigue, or chest pain; or improvement or cure as defined by the authors of the individual studies.	A meta-analysis using only the 5 RCTs demonstrated a pooled risk difference of 0.12 (95% confidence interval [CI], -0.04 to 0.20). This finding suggests that 12% of children treated with a macrolide will have more rapid clinical improvement, corresponding to a number needed to treat of 8.33, but the confidence interval overlapping 0% negates statistical significance. There remained significant heterogeneity between the studies (*P* = 0.02). The funnel plot revealed potential for publication bias against small studies that show a treatment effect.	The majority of studies included did not show a significant clinical benefit of M. pneumoniae spectrum therapy in CA-LRTI. Of the 9 studies that specifically examined the issue of M. pneumoniae treatment in children with CA-LRTI secondary to M. pneumoniae, almost all the prospective studies showed no clinical benefit. The remaining studies generally suggest a statistical, but not necessarily clinically relevant, decrease in fever duration, and most of these are rated as low- or lowest-quality evidence.	**Question 1** **Question 2**	
Antibiotics for community-acquired lower respiratory tract infections secondary to Mycoplasma pneumoniae in children Gardiner et al., 2015 [30]	1912 children (< 18 years of age) from 7 studies included studies for the qualitative evaluation	To determine whether antibiotics are effective in the treatment of childhood LRTI secondary to M. pneumoniae infections acquired in the community.	Primary outcomes 1. Proportions of participants who were not improved at follow-up. Secondary outcomes 1. Mean difference in symptoms and signs (mean improvement in clinical state). 2. Proportions requiring hospitalisation. 3. Proportions experiencing pulmonary complications (empyema, pleural effusion, air leak). 4. Proportions experiencing non-pulmonary complications. 5. Proportions experiencing adverse effects (for example, nausea, diarrhoea, abdominal pain, rash). 6. Proportions experiencing complications (for example, requirement for medication change).	This review failed to find any randomised controlled trials (RCTs) that specifically looked at the effectiveness of antibiotics for lower respiratory tract infection (LRTI) secondary to *M. pneumoniae*. In most studies, clinical response did not differ between children randomised to a macrolide antibiotic and children randomised to a non-macrolide antibiotic.	There is insufficient evidence to draw any specific conclusions about the efficacy of antibiotics for this condition in children (although one trial suggests macrolides may be efficacious in some children with LRTI secondary to *Mycoplasma)*. The use of antibiotics has to be balanced with possible adverse events.	**Question 1** **Question 2**	
Antibiotics for community-acquired pneumonia in children Lodha et al., 2013 [31]	14.188 children (< 18 years of age) from 29 included trials (Only 7 articles conducted in high income countries)	To identify effective antibiotic drug therapies for CAP of varying severity in children by comparing various antibiotics.	Clinical Cure and Treatment failure rates Secondary outcomes: relapse rate, hospitalization rate, length of hospital stay, need for change in antibiotics, additional interventions used, mortality rate	In ambulatory settings, for treatment of World Health Organization (WHO) defined non-severe CAP, amoxycillin compared with co- trimoxazole had similar failure rates (odds ratio (OR) 1.18, 95% confidence interval (CI) 0.91 to 1.51) and cure rates (OR 1.03, 95% CI 0.56 to 1.89). Three studies involved 3952 children.	For treatment of patients with CAP in ambulatory settings, amoxycillin is an alternative to cotrimoxazole. With limited data on other antibiotics, co-amoxiclav acid and cefpodoxime may be alternative second-line drugs. Children with severe pneumonia without hypoxaemia can be treated with oral amoxycillin in an ambulatory setting.	**Question 3** **Question 4**	
Antibiotic Treatment Duration for Community-Acquired Pneumonia in Outpatient Children in High-Income Countries—A Systematic Review and Meta-Analysis Kuitunen et al., 2022 [32]	4 RCTs, all conducted in high income countries (SCOUT-CAP, CAP-IT, SAFER, Greenberg et al.) 1541 children aged ≥ 6 months with CAP	To compare short antibiotic treatment (3–5 days) with longer treatment (7–10 days)	The need for antibiotic retreatment, hospitalization, or treatment failure (including either need for retreatment or hospitalization) within 1 month after the randomization. Secondary outcomes were antibiotic-related adverse effects.	All 4 studies assessed treatment failures, and the risk differences (RD) was 0.1% (95% confidence interval, − 3.0–2.0%) with high quality of evidence. Two studies (1194 children) assessed adverse events related to antibiotic treatment, and the RD was 0.0% (− 5.0–5.0%) with moderate quality of evidence	A short antibiotic treatment duration of 3–5 days was equally effective and safe compared with the longer (current) recommendation of 7–10 days in children aged ≥ 6 months with CAP. Short antibiotic courses can be implemented in treatment of pediatric CAP.	**Question 6**	
Short-Course vs. Long-Course Antibiotic Therapy for Children With Nonsevere Community-Acquired Pneumonia A Systematic Review and Meta-analysis Li et al., 2022 [33]	Nine randomized clinical trials (4 from high income countries, SCOUT-CAP, CAP-IT, SAFER, Greenberg et al., the same included in the review published by Kuitunen; 5 from low and middle income countries, Glinsburg et al., ISCAP, Kartasasmita, MASCOT, Lupisan) 11,143 children with CAP	To determine whether a shorter course of antibiotics was noninferior to a longer course for childhood non-severe CAP	Treatment failure, defined by persistence of pneumonia or the new appearance of any general danger signs of CAP (e.g., lethargy, unconsciousness, seizures, or inability to drink), elevated temperature (> 38 °C) after completion of treatment, change of antibiotic, hospitalization, death, missing more than 3 study drug doses, loss to follow-up, or withdrawal of informed consent.	Treatment failure occurred in 12.8% vs. 12.6% of participants randomized to a shorter vs. a longer course of antibiotics. Shorter course of oral antibiotic was noninferior to a longer course with respect to treatment failure for children with nonsevere CAP (risk ratio, 1.01; 95%CI, 0.92–1.11; risk difference, 0.00; 95%CI, − 0.01 to 0.01; I2 = 0%). A 3-day course of antibiotic treatment was noninferior to a 5-day course for the outcome of treatment failure (risk ratio, 1.01; 95%CI, 0.91–1.12; I2 = 0%), and a 5-day course was noninferior to a 10-day course (risk ratio, 0.87; 95%CI, 0.50–1.53; I2 = 0%). A shorter course of antibiotics was associated with fewer reports of gastroenteritis (risk ratio, 0.79; 95%CI, 0.66–0.95) and lower caregiver absenteeism (incident rate ratio, 0.74; 95%CI, 0.65–0.84).	A shorter course of antibiotics was noninferior to a longer course in children aged 2 to 59 months with nonsevere CAP. Clinicians should consider prescribing a shorter course of antibiotics for the management of pediatric nonsevere CAP. High-quality evidence from the review showed that in high income countries a 5-day regimen might be sufficient for the management of children with CAP	**Question 6**	
Shorter Versus Longer-term Antibiotic Treatments for Community-Acquired Pneumonia in Children: A Meta-analysis Gao et al., 2023 [34]	Sixteen trials (9 from high income countries, CAP-IT, Gomez Campdera 1996, Greenberg, Harris 1998, SAFER, Ronchetti 1994, Roord 1996, SCOUT-CAP, Wubbel 1999) 7 from low income countries, from 1994 to 2022, 1 conference abstract) 12 774 children with CAP under 18 years of age with diagnosed CAP according to investigator-defined definitions treated as outpatients with oral antibiotics	To compare the efficacy and safety of shorter versus longer duration of antibiotic treatment	Clinical cure, treatment failure, relapse, duration of hospital stay, mortality, need for change in antibiotics, ICU admission, duration of hospital stay, duration of ICU stay, hospital readmission, invasive ventilation, for trials enrolling outpatients the need for hospitalization, severe adverse events, and all adverse events.	Considering the high income countries, there are probably no substantial differences between shorter-duration and longer duration antibiotics in clinical cure (odds ratio 1.29, 95% confidence interval [CI] 0.95 to 1.77; moderate certainty), treatment failure (relative risk [RR] 0.82, 95% CI 0.48 to 1.40; moderate certainty), and relapse (RR 0.99, 95% CI 0.45 to 2.17; moderate certainty). Compared with longer-duration antibiotics, shorter-duration antibiotics do not appreciably increase mortality (RD 0.0%, 95% CI 0.2 to 0.1; high certainty), and probably have little or no impact on the need for hospitalization moderate certainty), and severe adverse events	Duration of antibiotic therapy likely makes no important difference in patient important outcomes.	**Question 6**	
Shorter versus longer duration of Amoxicillin‑based treatment for pediatric patients with community‑acquired pneumonia: a systematic review and meta‑analysis Marques et al., 2022 [35]	Three RCTs (SCOUT-CAP, SAFER, Greenberg) 789 children older than 6 months with CAP in an outpatient setting	To compare 5-day and 10-day courses of Amoxicillin	The outcome of interest was clinical cure	No differences were found between 5-day and 10-day therapy regarding clinical cure (RR 1.01; 95% CI 0.98–1.05; *p* = 0.49; I2 = 0%). Subgroup analysis of children aged 6–71 months showed no difference in the rates of the same outcome (RR 1.01; 95% CI 0.98–1.05; *p* = 0.38; I2 = 0%).	A short course of Amoxicillin (5 days) is just as effective as a longer course (10 days) for uncomplicated CAP in children under 10 years old. Nevertheless, generalizations should be made with caution considering the socioeconomic settings of the studies included.	**Question 6**	
**1b. RCT and Observational Studies**							
**Title, author, year of publication**	**Study design**	**Population** **(** ***N*** **°, Country, setting)**	**Exposure**	**Primary outcome**	**Follow-up**	**Results**	**Contributing Evidence**
Comparative Effectiveness of Beta-lactam vs. Macrolide monotherapy in Children with Pneumonia Diagnosed in the Outpatient Setting Ambroggio et al., 2015 [42]	Retrospective cohort study	*N* = 1,999 children with CAP treated in the outpatient setting, of whom 1,164 were matched in the treatment group. USA Children from 1 to 18 years of age with uncomplicated CAP	Beta-lactam or macrolide monotherapy.	Treatment failure, defined as a follow-up visit with an ICD-9 code for a respiratory-related diagnosis accompanied by a change in antibiotic therapy either in the outpatient setting (in-person or via phone), in the emergency department, or as a hospital admission	14-days	Patients who received macrolide monotherapy had no statistical difference in treatment failure regardless of age when compared with patients who received beta-lactam monotherapy. Among children younger than 6 years, there was no statistically significant difference in treatment failure within 14 days between those receiving beta-lactam monotherapy and those receiving macrolide monotherapy (Adjusted Odds Ratio (AOR): 0.90; 95% Confidence Interval: 0.37, 2.22)). Among those who were 6 years of age and older, children who received macrolide monotherapy had a non-statistically significant lower odds of treatment failure within 14 days compared with children 6 years of age and older who received beta-lactam monotherapy (AOR: 0.48; 95% CI: 0.22, 1.01).	**Question 1** **Question 2** **Question 4**
Beta-Lactam Versus Beta-Lactam/Macrolide Therapy in Pediatric Outpatient Pneumonia Ambroggio et al., 2016 [44]	Retrospective cohort study	*N* = 717 children with uncomplicated CAP treated in the outpatient setting. USA Children from 1 to 18 years of age	Beta-lactam monotherapy or beta- lactam/macrolide combination therapy	Treatment failure, defined as a follow-up visit within 14 days of diagnosis resulting in a change in antibiotic therapy.	14-day	Of 717 children in the analytical cohort, 570 (79.4%) received beta-lactam monotherapy and 147 (20.1%) received combination therapy. Of those who received combination therapy 58.2% of children were under 6 years of age. Treatment failure occurred in 55 (7.7%) children, including in 8.1% of monotherapy recipients, and 6.1% of combination therapy recipients. Treatment failure rates were highest in children 6–18 years receiving monotherapy (12.9%) and lowest in children 6–18 years receiving combination therapy (4.0%). Children 6–18 years of age who received combination therapy were less likely to fail treatment than those who received beta-lactam monotherapy (propensity-adjusted odds ratio, 0.51; 95% confidence interval, 0.28, 0.95).	**Question 1** **Question 2**
Effects of clinical pathway implementation on antibiotic prescriptions for pediatric community-acquired pneumonia Donà et al., 2018 [45]	Before and after	97 children with non-complicated CAP evaluated in PED Italy	CP for CAP to increase the use of narrow spectrum antibiotics and reduce the days of therapy	Change in antibiotics prescriptions and treatment duration Treatment failure, prescription of broad-spectrum antibiotics	30 days	Before implementation 50% of children (28/56) received exclusively amoxicillin, compared with 73.2% (30/41) after CP release. Pre-intervention median LOT was 10 (range 3–15), while post-intervention median LOT was 8 (range 5–10) (*p* < 0.0001) as recommended in the CP, with a decreasing trend over all sub-periods after implementation. In the pre-CP period, treatment failure occurred in 2.3% (1/44) of cases, while 11.8% (4/34) failed treatment in the post-CP period (*p* = 0.29)	**Question 1** **Question 2** **Question 6**
Antibiotic Choice and Clinical Outcomes in Ambulatory Children with Community- Acquired Pneumonia Lipsett et al., 2021 [46]	Retrospective cohort study	*N* = 252.177 outpatient pneumonia visits USA Children from 0 to 18 years of age diagnosed with CAP from 2010 to 2016	Narrow-spectrum (aminopenicillins), broad-spectrum (amoxicillin/clavulanate and cephalosporins), macrolide monotherapy, macrolides with narrow-spectrum antibiotics, or macrolides with broad-spectrum antibiotics.	Hospitalization, development of severe pneumonia and change in antibiotic therapy	7 days	Among 252,177 outpatient pneumonia visits, macrolide monotherapy was used in 43.2%, narrow-spectrum antibiotics in 26.1%, and broad-spectrum antibiotics in 24.7%. A total of 1488 children (0.59%) were subsequently hospitalized and 117 (0.05%) developed severe pneumonia. Compared with children receiving narrow-spectrum antibiotics, the odds of subsequent hospitalization were higher in children receiving broad-spectrum antibiotics (aOR = 1.34 [95%CI 1.17–1.52]) and lower in children receiving macrolide monotherapy (aOR = 0.64 [95%CI 0.55–0.73]) and macrolides with narrow-spectrum antibiotics (aOR = 0.62 [95%CI 0.39–0.97]). Children receiving macrolide monotherapy had lower odds of developing severe pneumonia than children receiving narrow-spectrum antibiotics (aOR = 0.56, 95%CI 0.33–0.93). However, the absolute risk difference was < 0.5% for all analyses.	**Question 1** **Question 2** **Question 4**
Comparative Effectiveness of Empiric Antibiotics for Community-Acquired Pneumonia Queen, 2014 [49]	OR	492 children, USA, inpatient, uncomplicated CAP	Empiric treatment with narrow- spectrum therapy versus broad-spectrum therapy	LOS Treatment failure, duration of fever and oxygen therapy	7-days	Narrow-spectrum therapy was not inferior to broad-spectrum antibiotics in all measured outcomes including LOS, duration of oxygen, duration of fever, daily standardized pharmacy and overall costs, or readmission rates within 7 days.	**Question 1** **Question 2**
Narrow Vs Broad-spectrum Antimicrobial Therapy for Children Hospitalized With Pneumonia Williams, 2013 [51]	OR	15,564 Children > 6 months, vaccinated, USA, inpatient, uncomplicated CAP	Empiric treatment with narrow- spectrum therapy versus broad-spectrum therapy	LOS Admission to intensive care (after the first 2 calendar days), 14- day all-cause readmission, and total costs for the admission and the entire episode of illness (accounting for 14-day readmissions)	14 days	There was no difference in length of stay, admission in PICU or readmission within 14 days of discharge	**Question 1** **Question 2**
Management of Pediatric Pneumonia: A Decade After the Pediatric Infectious Diseases Society and Infectious Diseases Society of America Guideline Ambroggio et al., 2023 [48]	Quasi-experimental study	Children aged 3 months–18 years with CAP who visited 1 of 28 participating hospitals in USA from 2009 to 2021 USA 315 384 children with CAP, 71 804 (22.8%) were hospitalized	Impact of IDSA guideline on the use of aminopenicillin in children with CAP treated as outpatient and inpatient	Change in antibiotic prescription For children hospitalized: hospital length of stay (LOS) in days, admission to an ICU, death during hospitalization, and readmission to the hospital within 7, 14, and 30 days. For children discharged from the ED: revisits occurring within 7, 14, and 30 days.	30 days	Among hospitalized children, there was an increase in aminopenicillin prescribing (1.1% per quarter). Among children discharged from the emergency department (ED), there was an increase in aminopenicillin prescription (0.45% per quarter). Hospital length of stay, ED revisit rates, and hospital readmission rates remained stable.	**Question 1** **Question 2**
Evaluation of a Pediatric Community-Acquired Pneumonia Antimicrobial Stewardship Intervention at an Academic Medical Center Puzz et al., 2023 [53]	Before and after	540 patients Children admitted for mild-moderate CAP during three time periods (pre-intervention and post-intervention groups 1 and 2) USA (Missisipi)	Local pediatric CAP treatment guidelines, and antimicrobial stewardship pharmacists, handshake stewardship (prospective audits with feedback and rounding in person)	Changes in inpatient antibiotic selection and duration following the interventions Discharge antibiotic regimens, length of stay, and 30-day	30 days	Antibiotic selection significantly improved, with prescriptions for ceftriaxone decreasing (*p* < 0.001) and ampicillin increasing (*p* < 0.001) following the interventions. Azithromycin prescribing also significantly decreased from the pre-intervention group to the post-intervention groups for monotherapy and combination therapy. Antibiotic duration decreased from a median of ten days in the pre-intervention group and post-intervention group 1 to eight days in post-intervention group 2. Length of stay decreased from a mean of 4.9 days to a mean of 2.07 and 1.92 in group 1 and 2 respectively. 30-day readmission was stable during the 3 periods (2%, 7%, 5% respectively)	**Question 1** **Question 2** **Question 6**
Effectiveness of β-Lactam Monotherapy vs. Macrolide Combination Therapy for Children Hospitalized With Pneumonia Williams et al., 2017 [52]	OB (multicenter retrospective cohort study)	1418 children (693 girls and 725 boys) children (up to 18 years of age) who were hospitalized with radiographically confirmed pneumonia USA	β-lactam monotherapy (oral or parenteral second- or third-generation cephalosporin, penicillin, ampicillin, ampicillin-sulbactam, amoxicillin, or amoxicillin-clavulanate) vs. β-lactam plus macrolide combination therapy	LOS Intensive care admission, rehospitalizations, and self-reported recovery at follow-up.	3 to 10 weeks following hospital discharge	In the unmatched cohort, there was no statistically significant difference in length of hospital stay between children receiving β-lactam monotherapy and combination therapy (median, 55 vs. 59 h; adjusted hazard ratio, 0.87; 95%CI, 0.74–1.01). The propensity-matched cohort (*n* = 560, 39.5%) showed similar results. There were also no significant differences between treatment groups for the secondary outcomes.	**Question 1** **Question 2**
Comparative Effectiveness of Empiric b-Lactam Monotherapy and b-Lactam–Macrolide Combination Therapy in Children Hospitalized with Community-Acquired Pneumonia Ambroggio et al., 2012 [43]	OB (multicenter retrospective cohort study)	20,743 children aged 1–18 years who were hospitalized with CAP USA	Empiric β-lactam monotherapy monotherapy (aminopenicillins, penicillin, second- and third-generation cephalosporins) versus β-lactam-macrolide combination therapy	LOS Readmission within 14 days of the index hospital discharge	14 days	Compared with children who received b-lactam monotherapy, children who received b-lactam plus macrolide combination therapy were 20% less likely to stay in the hospital an additional day (adjusted relative risk 0.80; 95% CI, 0.75–0.86) but did not have a different readmission rate (relative risk 0.69; 95% CI, 0.41–1.12). An effect of combination treatment on reduced length of stay was not evident in children < 6 years of age but increased with increasing age groups thereafter.	**Question 1 Question 2**
Impact of a Guideline on Management of Children Hospitalized With Community-Acquired Pneumonia Newman et al., 2012 [50]	Before and after	1033 children, USA, inpatient, uncomplicated CAP (vaccine status not known)	clinical practice guideline (CPG) for uncomplicated CAP: use of narrow-spectrum antibiotics (ampicillin, amoxicillin) for 5–7 days	Impact on antibiotic management in children hospitalized with uncomplicated CAP Treatment failure	30 days	Before the CPG, 13% of patients empirically received ampicillin and 72% received ceftriaxone. In the year after the CPG, 63% empiric received ampicillin and 21% received ceftriaxone. Overall, 8 (1.5%) pre- CPG patients and 5 (1%) post-CPG met failure criteria (*P* = 0.28).	**Question 3**
Antibiotic Treatment of Children With Community-Acquired Pneumonia: Comparison of Penicillin or Ampicillin Versus Cefuroxime Dinur-Schejter et al., 2012 [54]	OR	319 children, 3 months- 2 years, Israel, Inpatient, not complicated CAP, NOT vaccinated Further, they analyzed children with not complicated CAP 3 months-6 years	1° center IV cefuroxime (100 mg/kg/24 hr tid) 3 months-2 years 2° center IV penicillin (400,000 IU/kg/24 hr qid) or ampicillin (100–200 mg/kg/24 hr tid-qid)	LOS Treatment failure, duration of fever and oxygen therapy	None	Treatment outcomes were similar between the penicillin or ampicillin group and the cefuroxime group. The number of patients with treatment failure (defined as requiring a change of first-line treatment) was also similar between the two groups (7.6% vs. 4.7%). There were no difference also for children between 3 months and 6 years	**Question 3**
Amoxicillin duration and dose for community-acquired pneumonia in children: the CAP-IT factorial non-inferiority RCT Bielicki et al., 2021 [38]	Multicentre randomised double-blind 2 × 2 factorial non-inferiority trial	824 children aged > 6 months, weighing 6–24 kg, with a clinical diagnosis of community-acquired pneumonia	Oral amoxicillin syrup at a dose of 35–50 mg/kg/day compared with a dose of 70–90 mg/kg/day, and 3 compared with 7 days’ duration.	Any clinically indicated systemic antibacterial treatment prescribed for respiratory tract infection (including community-acquired pneumonia), other than trial medication, up to 28 days after randomisation. Phenotypic resistance to penicillin at day 28 measured in nasopharyngeal S. pneumoniae isolates, severity and duration of parent/guardian-reported CAP symptoms (including fever, cough, phlegm, fast breathing, wheeze, disturbed sleep, eating/drinking less, interference with normal activity and vomiting), adherence to trial medication, the occurrence of specified clinical AEs (including skin rash, thrush and diarrhoea) and serious adverse events (SAEs)	28 days	The observed number of primary end points was similar in the lower-dose arm (*n* = 51, 12.6%) and in the higher-dose arm (*n* = 49, 12.4%). The estimated risk difference at day 28 was 0.2% (90% CI − 3.7–4.0%), meeting the criterion for non-inferiority Of the 14 prespecified secondary end points, the only significant differences were 3-day vs. 7-day treatment for cough duration (median 12 days vs. 10 days; hazard ratio [HR], 1.2 [95% CI, 1.0 to 1.4]; *P* = 0.04) and sleep disturbed by cough (median, 4 days vs. 4 days; HR, 1.2 [95% CI, 1.0 to 1.4]; *P* = 0.03	**Question 5** **Question 6**
Short versus prolonged-duration antibiotics for outpatient pneumonia in children Shapiro et al., 2021 [47]	Retrospective cohort study	*N* = 121,846 Children from 1 to 18 years of age with outpatient CAP who filled a prescription for oral antibiotics USA	Short-vs prolonged-duration antibiotics	To determine associations between the duration of prescribed antibiotics (5–9 days vs. 10–14 days) and subsequent hospitalizations, new antibiotic prescription, and acute care visits.	14 days following the end of the dispensed antibiotic course	The most commonly prescribed duration of antibiotics was 10 days (82.8% of prescriptions), and 10.5% of patients received short-duration therapy. During the follow-up period, 0.2% of patients were hospitalized, 6.2% filled a new antibiotic prescription, and 5.1% had an acute care visit. Compared with the prolonged-duration group, the aORs for hospitalization, new antibiotic prescriptions, and acute care visits in the short-duration group were 1.16 (95% CI 0.80–1.66), 0.93 (95% CI 0.85–1.01), and 1.06 (95% CI 0.98–1.15), respectively.	**Question 6**
Short-Course Antimicrobial Therapy for Pediatric Community-Acquired Pneumonia. The SAFER Randomized Clinical Trial Pernica et al., 2021 [39]	2-center, parallel-group, blinded, noninferiority randomized clinical	281 Children aged 6 months to 10 years with CAP well enough to be treated as outpatients	Five days of high-dose amoxicillin therapy followed by 5 days of placebo (intervention group) vs. 5 days of high-dose amoxicillin followed by a different formulation of 5 days of high-dose amoxicillin (control group).	Clinical cure at 14 to 21 days. Number of days the participant was absent from school or daycare, the total number of days that caregiver work was disrupted, the number of days of mild adverse reactions to the drug, the incidence of serious adverse reactions to the drug (including anaphylaxis), participant adherence to the study medications, and recurrence of presumed bacterial respiratory illness after the primary outcome visit in the month after enrollment; post hoc, clinical cure not requiring additional intervention	21 days	Clinical cure was observed in 101 of 114 children (88.6%) in the intervention group and in 99 of 109 (90.8%) in the control group in per-protocol analysis (risk difference, − 0.016; 97.5% confidence limit, − 0.087). Clinical cure at 14 to 21 days was observed in 108 of 126 (85.7%) in the intervention group and in 106 of 126 (84.1%) in the control group in the intention-to-treat analysis (risk difference, 0.023; 97.5% confidence limit, − 0.061).	**Question 6**
Short- vs. Standard-Course Outpatient Antibiotic Therapy for Community-Acquired Pneumonia in Children; The SCOUT-CAP Randomized Clinical Trial (Williams, 2022, RCT) [40]	Multicenter randomized double-blind placebo-controlled superiority clinical trial	385 healthy children aged 6 to 71 months with nonsevere CAP demonstrating early clinical improvement	On day 6 of their originally prescribed therapy, participants were randomized 1:1 to receive 5 days of matching placebo or 5 additional days of the same antibiotic.	End-of-treatment response adjusted for duration of antibiotic risk (RADAR), a composite end point that ranks each child’s clinical response, resolution of symptoms, and antibiotic-associated adverse effects in an ordinal desirability of outcome ranking (DOOR) Treatment failure RADAR at OAV2 as well as DOOR and its components (adequate clinical response, resolution of symptoms, and antibiotic-associated adverse effects) at OAV1 and OAV2.	25 days OAV1 6–10 days OAV2 19–25 days	A 5-day antibiotic strategy was superior to a 10-day strategy. There were no significant differences between treatment strategies in proportions of inadequate clinical response at OAV1 (1% vs. < 1%; difference in proportion, 0.5%; 95% CI, − 2.4 to 3.7) or OAV2 (1% vs. 2%; difference in proportion, − 0.5%; 95% CI, − 3.9 to 2.8) When duration of antibiotic treatment was incorporated, the short-course strategy was superior, with an estimated probability of a more desirable RADAR for the short-course strategy of 0.69 (95% CI, 0.63–0.75) at OAV1. At OAV2, the probability of a more desirable RADAR in the short-course strategy was 0.63 (95% CI, 0.57–0.69).	**Question 6**
Short-course Antibiotic Treatment for Community-acquired Alveolar Pneumonia in Ambulatory Children. A Double-blind, Randomized, Placebo-controlled Trial Greenberg, 2014, RCT [41]	A double-blind, randomized, placebo-controlled trial	140 healthy children aged 6 to 59 months not vaccinated with non severe CAP	Stage 1: 3-day course vs. 10-day course of amoxicillin treatment Stage 2: 5-day course vs. 10-day course.	Absence of treatment failure within 30 days Temperature, difficult breathing, restlessness, coughing, loss of appetite and sleep disturbances assessed daily by the parents; laboratory tests: complete WBC counts and CrP at days 5–7 and 10–14	30 days	3-day oral treatment was associated with high treatment failure 5-day oral treatment with high-dose amoxicillin (80 mg/kg/d divided to 3 daily doses) is as effective as a 10-day treatment in children 6–59 months of age with nonsevere CAP	**Question 6**
Antibiotic Treatment for Children Hospitalized With Community-Acquired Pneumonia After Oral Therapy Breuer et al., 2015 [55]	Retrospective observational study	All children aged 3 months-18 years with uncomplicated CAP who received oral antibiotics prior to admission	Narrow spectrum antibiotics (penicillin, ampicillin, amoxicillin) versus broad spectrum antibiotics (ceftriaxone, cefuroxime, cefazolin)	Clinical outcome of previously healthy children with non-complicated CAP Duration of fever, duration of intravenous (IV) antibiotic therapy, and total hospital length of stay (LOS) Treatment failure (defined as a change in the antibiotic therapy after the first 24 h of treatment) and number of days of oxygen treatment.	Not specified	The broad spectrum-treated group had significantly better outcomes in terms of number of febrile days (1.2 ± 1.1 vs. 1.7 ± 1.6, *p* < 0.001), number of days treated with intravenous antibiotics (3.1 ± 1.3 vs. 3.9 ± 2.0, *p* < 0.001), and days of hospitalization (3.5 ± 1.5 vs. 4.2 ± 2.0, *p* < 0.001). The odds ratio for remaining hospitalized at 72 h and 7 days was significantly higher for the narrow spectrum group (2.0 and 5.5 respectively, *p* < 0.05)	**Question 7**

No study was sponsored or funded by pharmaceutical companies

Overall, the studies were considered of low to moderate quality (Supplementary Material, Sects. 5, 6 and 7). Two reviews [36, 37] were subsequently excluded due to their low quality. The quality of evidence evaluated using the GRADE method was generally very low or low, only occasionally moderate (Supplementary Material, Sect. 8). The recommendations with the certainty of evidence by GRADE and the strength of each recommendation formulated considering both the evidence and the expert opinion panel members is reported in Table 2.

**Table 2:** Questions, recommendations and strength, quality of evidence, and Consensus panel

Question	Recommendation	Quality of evidence	Strength of the recommendation	Consensus panel
**1. What is the first-line antibiotic for treating mild-to-moderate CAP in a child under five years old with a complete immunization schedule (at least two doses of hexavalent and pneumococcal vaccines)?**	In children under five with a complete immunization schedule and mild-moderate CAP, amoxicillin prescription is recommended.	Low/very low	Strong recommendation in favor of the intervention	100%
**2. What is the first-line antibiotic for treating mild-moderate CAP in a child over five years old with a complete immunization schedule (at least two doses of hexavalent and pneumococcal vaccines)?**	In children over five years of age with a complete immunization schedule and mild-moderate CAP, treatment with amoxicillin is recommended.	Low/very low	Strong recommendation in favor of the intervention	100%
	In children who remain well-appearing and do not require hospitalization, macrolide therapy is recommended if there is no clinical improvement after 48 h of amoxicillin treatment.	Low/very low	Strong recommendation in favor of the intervention	100%
**3. What is the first line antibiotic for treating mild-moderate CAP in a child without a complete immunization schedule (< 2 doses of hexavalent and pneumococcal vaccines)?**	In children who are either unimmunized or have incomplete immunization against S. pneumoniae but are immunized for H. influenzae (having received more than two doses of hexavalent vaccine but less than two doses of pneumococcal vaccine) and present with mild-moderate CAP, monotherapy with amoxicillin is recommended as the first-line therapy.	Low	Strong recommendation in favor of the intervention	100%
	For children who are either unimmunized or have incomplete immunization coverage for both H. influenzae and S. pneumoniae (having received less than two doses of hexavalent and pneumococcal vaccines), first-line therapy with amoxicillin-clavulanate or second or third-generation cephalosporins is recommended.	Low	Weak recommendation in favor of the intervention	100%
**4. What is the first-line antibiotic in the treatment of mild-moderate bacterial CAP in patients allergic to penicillin?**	In patients with CAP and a suspected allergy to amoxicillin, who have not undergone allergological workup, the selection of alternative antibiotics (such as third-generation cephalosporins or macrolides) should be guided by meticulous risk stratification.	Very low	Strong recommendation in favor of the intervention	100%
	In patients with CAP suspected of having an allergy to amoxicillin and deemed to be at low risk of allergic reaction, a second or third-generation cephalosporin (such as cefuroxime or cefpodoxime proxetil) is recommended as an alternative therapy. The utilization of macrolides (like clarithromycin) or clindamycin should be reserved for patients at high risk of allergic reaction, with consideration given to levofloxacin for older children	Very low	Weak recommendation in favor of the intervention	100%
**5. What should be the optimal dosage of amoxicillin in treating mild to moderate bacterial CAP?**	To treat mild-moderate CAP, we recommend administering amoxicillin at a dosage of 80–90 mg/kg/day divided into three separate doses (with a maximum of 1 g three times a day). However, to enhance compliance with antibiotic therapy, particularly in cases of mild pneumonia with close clinical follow-up, the number of daily administrations can be reduced to two instead of three.	Moderate	Weak recommendation in favor of the intervention	100%
**Question 6. What should be the optimal length of therapy with amoxicillin for treating mild -moderate bacterial CAP?**	For the management of mild-moderate CAP, a 5-day course of antibiotic therapy with amoxicillin is recommended. Close clinical monitoring and reassessment are advised approximately 72 h after initiating antibiotic therapy to evaluate symptom resolution. If necessary, treatment may be extended for up to 7 days.	Moderate	Strong recommendation in favor of the intervention	100%
**7. What is the most appropriate antibiotic therapy in a child with CAP experiencing clinical deterioration after 48 h of first-line therapy with amoxicillin?**	In children experiencing clinical deterioration after 48 h of first-line therapy, hospitalization and treatment with broad-spectrum antibiotics are recommended.	Very low	Weak recommendation in favor of the intervention	100%

The following results and recommendations are mainly based on the outcome of “treatment failure” because a paucity of studies was found regarding the risk of developing resistance to antibiotics and the risk of adverse events associated with antibiotic therapy without significant results.

**Question 1. What is the first-line antibiotic for treating mild-to-moderate CAP in a child under five years old with a complete immunization schedule (at least two doses of hexavalent and pneumococcal vaccines)?**

### Recommendation 1

**In children under five with a complete immunization schedule and mild-moderate CAP**,** amoxicillin prescription is recommended. ***(Very low quality of evidence for amoxicillin vs. broad-spectrum antibiotics*,* amoxicillin vs. macrolides*,* beta-lactams vs. beta-lactams plus macrolides*,* aminopenicillin vs. broad-spectrum antibiotics; low quality of evidence for beta-lactams vs. macrolides. Strong recommendation in favor of the intervention. 100% panel consensus)*.

From the analysis of the collected scientific evidence (Table 1), it has been suggested that therapy with narrow-spectrum antibiotics such as amoxicillin and intravenous aminopenicillins is not, in most cases, inferior in terms of treatment failure risk compared to therapy with broad-spectrum antibiotics (such as amoxicillin-clavulanate or 2nd or 3rd generation cephalosporins) or combination therapy of beta-lactams with macrolides [42–45, 48, 49, 51–53] (very low quality of evidence for amoxicillin vs. broad-spectrum antibiotics, amoxicillin vs. macrolides, beta-lactams vs. beta-lactams plus macrolides, aminopenicillin vs. broad-spectrum antibiotics; low quality of evidence for beta-lactams vs. macrolides).

In the study published by Ambroggio et al. in 2015 on a cohort of 1164 pediatric patients with pneumonia treated as outpatient, no statistically significant difference in treatment failure at 7 and 14 days was found between the group treated with beta-lactams (penicillins, aminopenicillins and third generation cephalosporins) and that treated with macrolides [Adjusted Odds Ratio (AOR): 0.90; 95% Confidence Interval (CI): 0.37–2.22] [42]. The same result was observed comparing children treated with beta-lactams to those treated with the combination therapy of beta-lactams and macrolides (cohort of 717 children, treatment failure at 7 days OR 1.33, 95%CI 0.74–2.39 and at 14 days OR 1.34, 95%CI 0.83–2.18) [44]. Instead, the study published by Lipsett et al. in 2021, on a cohort of 252,177 children with non-severe community-acquired pneumonia treated as outpatient, showed slightly different results, as combination therapy with beta-lactams (both narrow and broad spectrum) plus macrolides showed a reduction in the number of new antibiotics prescriptions compared to beta-lactams alone [narrow-spectrum plus a macrolide vs. narrow-spectrum (OR 0.47, 95% CI: 0.39–0.57); broad-spectrum plus a macrolide vs. broad-spectrum (OR 0.48, 95% CI: 0.41–0.56)]. On the contrary, an increase in the number of new antibiotic prescriptions was observed comparing the group treated with broad-spectrum antibiotics compared to the narrow-spectrum one (OR 1.15; 95% CI: 1.09–1.21), whereas no difference was observed comparing children treated with narrow-spectrum and those treated with macrolides (OR 1.15; 95% CI: 1.09–1.21) [46]. Nevertheless, this study included children aged 1 month to 18 years without stratifying the analysis by age group. This may have introduced a bias, as older children could benefit more from macrolide therapy for atypical pneumonia.

Different systematic reviews report on the treatment of microbiologically confirmed pneumonia caused by *M. pneumoniae*. No statistically significant differences were observed in patients treated with macrolides compared to those treated with beta-lactams [29, 30].

The systematic review with meta-analysis by Biondi et al., published in 2015, included 16 articles, published between 1967 and 2012, regarding the treatment of microbiologically confirmed pneumonia caused by Mycoplasma pneumoniae, without age distinction. Only five studies were included in the meta-analysis, showing a risk difference of 0.12 (95%CI, -0.04 to 0.20). Indeed, macrolides did not show a significant clinical benefit compared to beta-lactams.

Nevertheless, the studies included in the reviews presented significant heterogeneity, with the inclusion of a population with both lower and upper respiratory tract infections One of the studies under consideration documented a decrease in treatment failure risk among subjects administered macrolides [46]. However, these findings pertained to the broader pediatric population up to 18 years old, without specific disaggregation for those under five. This detail is pivotal as it’s recognized that macrolide treatment might be beneficial for older children, who are epidemiologically more prone to atypical pneumonia.

Indeed, atypical pneumonia may resolve spontaneously or benefit from macrolide therapy to support recovery. Nevertheless, while the authors aimed to balance comparison groups regarding pneumonia severity, it’s possible that children receiving macrolides had less severe pneumonia initially. It’s also important to recognize that a significant proportion of pediatric pneumonia cases are viral. Therefore, the increased efficacy observed in some groups of children treated with macrolides may be influenced more by the underlying causes than just the antibacterial effects.

The recommendation to use amoxicillin, albeit based on low-quality studies due to the retrospective observational nature of the studies and the diagnostic uncertainty that characterised all these studies, confirms what has been reported by the main guidelines published before 2012 by IDSA [8] the British Thoracic Society [9] and by a discussion paper published in collaboration with ESPID [10]. The guidelines rely on pre-2012 studies, excluded from this review due to agreed temporal constraints on article selection. The efficacy of amoxicillin in treating *Streptococcus pneumoniae* infections has long been established and substantiated scientifically. Amoxicillin stands as a preferred therapeutic option for bacterial infections caused by this pathogen, encompassing lung infections, otitis, and sinusitis, owing to its demonstrated efficacy, minimal side effects, and affordability [56–59]. This historical evidence, coupled with expert consensus, has enabled the formation of robust recommendations, even in light of limited or very low-quality evidence.

**Question 2. What is the first-line antibiotic for treating mild-moderate CAP in a child over five years old with a complete immunization schedule (at least two doses of hexavalent and pneumococcal vaccines)?**

### Recommendation 2

**In children over five years of age with a complete immunization schedule and mild-moderate CAP**,** treatment with amoxicillin is recommended. ***(Very low quality of evidence for amoxicillin vs. broad-spectrum antibiotics*,* aminopenicillin vs. broad-spectrum antibiotics. Strong recommendation in favor of the intervention. 100% panel consensus)*.

**In children who remain well-appearing and do not require hospitalization**,** macrolide therapy is recommended if there is no clinical improvement after 48 h of amoxicillin treatment. ***(Very low quality of evidence amoxicillin vs. macrolides*,* beta-lactams vs. beta-lactams pus macrolides; low quality of evidence for beta-lactams vs. macrolides. Strong recommendation in favor of the intervention. 100% panel consensus)*.

For children older than 5 years, it appears that combination therapy involving beta-lactams alongside macrolides may mitigate the risk of treatment failure compared to beta-lactam monotherapy [42, 44, 46], although other studies showed no difference in treatment failure [29, 30, 45, 48, 49, 51, 53] (Table 1). However, the evidence quality remains low due to the retrospective nature of most of the studies included, the uncertainty of the diagnosis and the considerable heterogeneity among study outcomes. Notably, one study documented a statistically significant reduction in hospitalization duration in moderate CAP cases treated with combination therapy, particularly in those over 6 years old, although no difference was noted in rehospitalization risk [43]. Conversely, another study failed to observe any significant difference in clinical improvement between children administered combination therapy versus monotherapy, prompting queries regarding the practical efficacy of macrolide antibiotic regimens, especially in hospitalized patients [52]. This observation is significant, given that atypical bacterial pneumonia cases often do not necessitate hospitalization and can resolve spontaneously. However, initiating specific antibiotic therapy may expedite recovery, potentially leading to a quicker resolution [52].

Given these results, it is recommended to consider starting macrolide in children over 5 years with CAP who have not shown clinical improvement after 48 h of amoxicillin treatment but remain well-appearing and do not require hospitalization.

This aligns with the IDSA guidelines [8], which recommend considering this therapy for children over 5 years old. Considering the growing prevalence of macrolide-resistant *S. pneumoniae* strains and the substantial rate of spontaneous eradication of *Mycoplasma pneumoniae* documented by certain studies before 2012, employing macrolides as first-line therapy is not deemed appropriate [60–62].

**Question 3. What is the first line antibiotic for treating mild-moderate CAP in a child without a complete immunization schedule (< 2 doses of hexavalent and pneumococcal vaccines)?**

### Recommendation 3

**In children who are either unimmunized or have incomplete immunization against *S. pneumoniae*but are immunized for *H. influenzae*type b (having received at least two doses of hexavalent vaccine but less than two doses of pneumococcal vaccine) and present with mild-moderate CAP**,** monotherapy with amoxicillin is recommended as the first-line therapy**. (Low-quality evidence. Strong recommendation in favor of the intervention. Panel consensus 100%)

**For children who are either unimmunized or have incomplete immunization coverage for both *H. influenzae*type b and *S. pneumoniae*(having received less than two doses of hexavalent and pneumococcal vaccines)**,** first-line therapy with amoxicillin-clavulanate or second or third-generation cephalosporins is recommended**. (Low-quality evidence. Weak recommendation in favor of the intervention. Panel consensus 100%)

Literature evidence concerning first-line therapy in unimmunized children is scarce, particularly in outpatient settings (Table 1). Studies focusing on unimmunized children primarily involve those hospitalized for non-severe pneumonia, often immunized for *H. influenzae* type b but not *S. pneumoniae*. Among the available studies, there’s an indication that oral narrow-spectrum antibiotic therapy is comparable in efficacy to intravenous therapy [50, 54, 63].

Regarding macrolide therapy, studies by Roord and Block (included in the meta-analysis published by Lodha [31]) suggested the effectiveness of these antibiotics in treating CAP in unimmunized children, considering the high prevalence of isolated atypical bacteria. However, two crucial aspects merit consideration: firstly, the presence of atypical bacteria on nasopharyngeal swabs does not definitively establish them as the causative agents of pneumonia and the presence of typical bacteria should also be taken into account. Secondly, these studies were conducted in the early 1990s when the prevalence of macrolide-resistant pneumococci was significantly lower than today, explaining the favourable response to these antibiotics even in cases of pneumonia caused by typical bacteria [64, 65]. Indeed, macrolide-resistant pneumococci were almost lower than 10% before 1990 worldwide, but it started to increase, reaching more than 20–30% in late 1990 [66]. Over time, the improper and excessive use of macrolides has led to the emergence of resistance mechanisms in *S. pneumoniae*, the primary pathogens responsible for CAP [67]. In 2022, in Italy, over 25% of *S. pneumoniae* strains were resistant to macrolides [66].

The quality of the available evidence is low, due to the observational nature of most of the studies and the uncertainty of the diagnosis, and there are no studies found specifically addressing subjects unimmunized for *H. influenzae* type b.

Given *S. pneumoniae*’s resistance mechanism to penicillin, attributed to the production of penicillin-binding proteins rather than beta-lactamases [68], a robust recommendation supports using amoxicillin in subjects immunized for *H. influenzae* type b but not for *S. pneumoniae*. Conversely, *H. influenzae*’s resistance mechanism to penicillin stems from the production of beta-lactamases [69]. Consequently, antibiotics coupled with inhibitors of these enzymes become necessary, as evidenced by studies on other infectious diseases like acute otitis media. However, since there is a dearth of studies directly involving patients unimmunized for *H. influenzae* type b, thus lacking comprehensive data on the actual incidence of pneumonia infections from this pathogen in such subjects, the recommendation for the use of amoxicillin-clavulanate in these patients is weak, primarily extrapolated from other pathologies such as acute otitis media.

**Question 4. What is the first-line antibiotic in the treatment of mild-moderate bacterial CAP in patients allergic to penicillin?**

### Recommendation 4

**In patients with CAP and a suspected allergy to amoxicillin**,** who have not undergone allergological workup**,** the selection of alternative antibiotics (such as third-generation cephalosporins or macrolides) should be guided by meticulous risk stratification.** (Very low-quality evidence. Expert opinion. Strong recommendation in favor of the intervention. Panel consensus 100%).

**In patients with CAP suspected of having an allergy to amoxicillin and deemed to be at low risk of allergic reaction**,** a second or third-generation cephalosporin (such as cefuroxime or cefpodoxime proxetil) is recommended as an alternative therapy. The utilization of macrolides (like clarithromycin) or clindamycin should be reserved for patients at high risk of allergic reaction**,** with consideration given to levofloxacin for older children** (Very low-quality evidence. Expert opinion. Weak recommendation in favor of the intervention. Panel consensus 100%).

Literature regarding penicillin allergy remains scarce (Table 1). β-lactam hypersensitivity reactions can be classified based on the time between antibiotic intake and symptom onset into immediate (symptoms within 1 h) and delayed (after 1 h). Immediate reactions are usually IgE-mediated and present as anaphylactic shock, angioedema, urticaria, and bronchospasm. Non-immediate reactions are typically non-IgE-mediated, with the most common clinical manifestation being a maculopapular rash, which is typically non-pruritic [70–73].

The risk of cross-reactivity between penicillin and cephalosporins remains a contentious issue [74]. Non-recent studies have reported a cross-reactivity frequency of 10% of cases. However, these studies were affected by evident biases, including the definition of allergy based solely on clinical history, the utilization of reference preparations of cephalosporins that were still “unrefined” and potentially contaminated by penicillin, and a lack of precise knowledge regarding the molecular structures of the compounds involved. Studies utilizing monoclonal antibodies have further revealed that for cephalosporins, the principal antigenic determinants are represented by the side chains. Therefore, in cases where the side chains differ from those of penicillin or amoxicillin, an increased risk of cross-reactions has not been demonstrated [75]. Supporting this, cefuroxime (a second-generation cephalosporin) has frequently been observed to be safe in patients with confirmed hypersensitivity to other beta-lactams, with cross-reactions occurring in only 6.3% of cases. More frequent cross-reactions have been noted with first-generation cephalosporins, which possess a beta-lactam ring similar to penicillins [76]. Given the low cross-reactivity observed in various studies, Drug Provocation Tests (DPT) are often conducted with cephalosporins featuring different side chains.

In non-IgE-mediated forms, T-lymphocytes play a role, and therefore, cross-reactivities are even rarer. In fact, 97.2% of individuals with hypersensitivity to aminopenicillins tolerate cephalosporins with different side chains, suggesting tolerability in the majority of cases [75]. Nonetheless, if penicillin allergy is confirmed, DPT with cephalosporins featuring different side chains may be considered.

In various allergy guidelines [70–73], there is no definitive consensus on the optimal method to ascertain if a patient with penicillin allergy can safely take a cephalosporin with different side chains directly or following DPT. However, the allergological assessment for a patient with penicillin allergy should ideally culminate in recommendations regarding the potential use of other beta-lactams, particularly cephalosporins. The determination of whether such use is feasible serves as guidance for family pediatricians in their future therapeutic prescribing practices.

Although macrolides therapy showed efficacy in treating CAP [31, 46, 64, 65], it’s important to note that most of these studies were conducted in the early 1990s when the prevalence of pneumococcal resistance to macrolides was significantly lower than it is today. One study in pediatric population showed that levofloxacin therapy was non-inferior to beta-lactam or macrolide therapy in terms of clinical cure rates, without difference in serious adverse events [61].

Considering the limited scientific evidence available, children with CAP and penicillin allergy, whether IgE or non-IgE mediated, may be treated with cephalosporins with different side chains (such as cefuroxime or cefpodoxime proxetil) only after undergoing an adequate allergological workup to assess their potential use. Alternatively, depending on the local prevalence of pneumococcal resistance to specific drug classes, clindamycin, clarithromycin, or, in older children, levofloxacin can be utilized [72].

**Question 5. What should be the optimal dosage of amoxicillin in treating mild to moderate bacterial CAP?**

### Recommendation 5

**To treat mild-moderate CAP**,** we recommend administering amoxicillin at a dosage of 80–90 mg/kg/day divided into three separate doses (with a maximum of 1 g three times a day). However**,** to enhance compliance with antibiotic therapy**,** particularly in cases of mild pneumonia with close clinical follow-up**,** the number of daily administrations can be reduced to two instead of three.** (Moderate quality of evidence. Weak recommendation in favor of the intervention. Panel consensus 100%)

The available data regarding the optimal dosage of amoxicillin for treating CAP are very limited (Table 1). The study conducted by Bielicki et al. [38] suggests the non-inferiority of low-dose therapy compared to high-dose therapy, administering the antibiotic twice a day instead of three times. Despite the robustness of the trial, intrinsic limitations may introduce bias. As the authors noted, it remains unclear how many enrolled children had bacterial pneumonia versus viral pneumonia that might have resolved spontaneously without antibiotic therapy. Although efforts were made to limit bias by excluding children with acute bronchospasm lacking clear signs of pneumonia, the uncertainty persists. Additionally, the median age of the participants was notably low, under three years, a period when most CAP cases are viral. In such instances, antibiotics may be unnecessary, suggesting that the lack of difference in treatment failure between the low and high dose groups could reflect the low incidence of bacterial pneumonia in this age group rather than the efficacy of the low dose.

Moreover, this study was conducted in a setting with a low prevalence of *S. pneumoniae* resistant to penicillin and amoxicillin. Since pneumococcal resistance to penicillin is attributed to genetic mutations that alter penicillin-binding protein structure, resulting in decreased affinity for all beta-lactam antibiotics, increasing the dosage of penicillin to saturate the binding sites becomes necessary to counteract this resistance mechanism. The other studies included in this consensus primarily report the use of high-dose amoxicillin administered in three doses for the treatment of community-acquired pneumonia [39, 41, 45]. However, these studies do not compare different dosages in terms of therapeutic efficacy and the risk of adverse effects It is therefore plausible that in regions with a low prevalence of resistance, therapy with reduced doses of amoxicillin may be equally effective compared to higher doses. However, considering the specific resistance mechanism, the differences in antimicrobial resistance between Northern and Southern Europe, and awaiting further studies, the panel’s decision in our region is to continue recommending therapy with high-dose amoxicillin (90 mg/kg/day), administered in three daily doses. Nevertheless, the strength of recommendation is weak, as lower dose might be efficacy as well. With close clinical monitoring, there is potential to reduce the number of daily administrations to two instead of three to enhance adherence to antibiotic therapy in cases of mild pneumonia.

**Question 6. What should be the optimal length of therapy with amoxicillin for treating mild -moderate bacterial CAP?**

### Recommendation 6

**For the management of mild-moderate CAP**,** a 5-day course of antibiotic therapy with amoxicillin is recommended. Close clinical monitoring and reassessment are advised approximately 72 h after initiating antibiotic therapy to evaluate symptom resolution. If necessary**,** treatment may be extended for up to 7 days**. (Moderate quality of evidence. Strong recommendation in favor of the intervention. Panel consensus 100%)

Based on the analysis of scientific evidence derived from four different randomized control trials [38–41], four systematic reviews [32–35], and three observational studies [45, 47, 53], it has been established that short-term therapy is not inferior to long-term therapy in treating mild-moderate CAP. Long-term therapy, extending to 10 days or beyond, does not appear to offer any advantage in terms of reducing therapeutic failures compared to shorter durations of therapy. Consequently, it is not recommended to prescribe prolonged antibiotic therapy, unless complete clinical resolution is not achieved during follow-up assessments.

The duration of short therapy varies across studies, ranging from 3 to 7 days. However, the sole trial that compared a 3-day therapy regimen to a 10-day regimen was suspended due to an increase in therapeutic failures [41], whereas no difference was observed comparing a 3-day therapy regimen to a 7-day therapy regimen [38].

Given the challenge of definitively determining how many of the enrolled children in various studies had bacterial pneumonia versus viral pneumonia, which could have resolved spontaneously without antibiotic therapy, it is recommended to maintain close follow-up approximately 72 h after initiating antibiotic therapy, by phone call in case of improvement and reliable family or re-evaluation in person in case of persistence of symptoms or unreliable family. This allows for assessment of the need to extend antibiotic therapy if symptoms do not completely resolve.

Therefore, until further studies with more robust scientific evidence comparing 3 and 5 days of therapy are available, the panel recommends prescribing antibiotic therapy for 5 days to children with uncomplicated CAP. Close clinical follow-up and reevaluation should occur approximately 72 h after initiating antibiotic therapy to assess symptom resolution or the necessity for continuation up to 7 days if needed.

**Question 7. What is the most appropriate antibiotic therapy in a child with CAP experiencing clinical deterioration after 48 h of first-line therapy with amoxicillin?**

### Recommendation 7

**In children experiencing clinical deterioration after 48 h of first-line therapy**,** hospitalization and treatment with broad-spectrum antibiotics are recommended.**

(Evidence quality very low. Weak recommendation in favor of the intervention. Panel consensus 100%)

No articles were identified in the literature regarding second-line therapy to be used in the community in the event of therapeutic failure and clinical deterioration. Several factors hindered the inclusion of additional studies comparing broad-spectrum and narrow-spectrum therapy in children hospitalized for CAP. Firstly, many of these studies also encompassed children with complicated pneumonia, a population not addressed in this consensus. Additionally, these studies often failed to specify whether hospitalization and the initiation of broad-spectrum parenteral antibiotic therapy were due to therapeutic failure of prior oral therapy or solely based on the child’s clinical condition. Consequently, it was challenging to understand whether children who had not improved with oral therapy were treated with narrow-spectrum or broad-spectrum therapy and what clinical improvement they had. Only one study was found regarding treatment failure of oral antibiotics, but children were hospitalized and treated with narrow-spectrum antibiotics (penicillin, ampicillin, amoxicillin) or broad-spectrum antibiotics (ceftriaxone, cefuroxime) with a statistically significant reduction in the duration of fever and length of stay in hospital in the broad-spectrum group [55].

Therefore, until further studies with more robust scientific evidence become available to recommend oral antibiotic therapy in cases of therapeutic failure associated with clinical deterioration, it is advised to hospitalize the child and initiate treatment with broad-spectrum antibiotics intravenously.

## Discussion

The diagnosis of CAP relies primarily on clinical assessment, which often introduces uncertainty, particularly in distinguishing between viral and bacterial infections, a challenge commonly faced by primary care pediatricians [6]. Despite this, there is significant room for improvement in antibiotic utilization [45, 48, 50, 53]. The optimal therapeutic approach to CAP remains a topic of ongoing debate. While the scientific community underscores the importance of employing amoxicillin as the first-line treatment for patients with suspected or confirmed mild-moderate CAP [8–10], this recommendation is frequently disregarded [77]. Instead, broader-spectrum antibiotics like amoxicillin-clavulanate, second or third-generation cephalosporins, or macrolides are often preferred [42, 44, 46]. Furthermore, there is still no consensus and few robust studies regarding the optimal dosage [38] and duration of therapy [32–35, 38–40, 47], underscoring the need for additional studies. Nevertheless, to enhance antibiotic stewardship in children with mild-moderate CAP treated as outpatients, we have developed this consensus after meticulous consideration of the best available evidence and extensive discussions with an expert panel.

Priorities for further studies should focus on several key areas. Firstly, improving diagnostic accuracy is crucial. Future research should aim to develop and refine diagnostic tools, including point-of-care tests, and methods to accurately distinguish between viral and bacterial CAP in clinical settings. This will help primary care pediatricians make more informed treatment decisions. Secondly, optimizing antibiotic utilization in CAP is essential. Studies should explore strategies to ensure that antibiotic treatments are both effective and aligned with best practices in antibiotic stewardship. Understanding the factors that lead to the frequent use of broader-spectrum antibiotics over recommended first-line treatments like amoxicillin is also important. Thirdly, there is a need for research on therapeutic approaches. Comparative studies should evaluate the efficacy of different antibiotic regimens, including the recommended first-line treatment with amoxicillin versus broader-spectrum antibiotics. These studies should aim to establish clear guidelines on the most effective therapeutic approaches for mild to moderate CAP in children. Fourthly, determining the optimal dosage and duration of antibiotic therapy for CAP is critical. Further evidence are needed to establish treatment protocols that minimize side effects and resistance while maximizing therapeutic outcomes. Lastly, research should address the barriers to the implementation of evidence-based guidelines in clinical practice. This includes understanding why first-line treatment recommendations are often disregarded and developing strategies to improve adherence among healthcare providers. By addressing these priorities, future studies can significantly contribute to better management of CAP in children, ensuring that treatments are both effective and responsible in terms of antibiotic use.

Our study has some limitations. As already mentioned above, the diagnosis of CAP is primarily clinical and not always definitive. The studies included in the analysis may have enrolled subjects diagnosed with CAP using various diagnostic methods, ranging from those with radiologically confirmed pneumonia to those diagnosed based solely on clinical presentation such as fever and respiratory symptoms. Furthermore, within the same study, individuals with bacterial CAP may have been enrolled alongside those with viral CAP, for whom antibiotic therapy would not be necessary. Additionally, different studies may have assessed outcomes in varied ways. For instance, treatment failure might have been evaluated based on the need for a new antibiotic prescription, hospitalization, admission to the intensive care unit, duration of fever, or achievement of clinical recovery within different timeframes specified by each study. However, the definition of clinical recovery itself may not have been consistent across studies, further complicating the interpretation of the already limited evidence.

It’s crucial to highlight that the temporal limitation imposed, focusing solely on studies published from 2012 onwards, primarily emphasized more recent and supported topics such as therapy duration. However, this came at the inevitable expense of studies investigating the selection of the most appropriate antibiotic, a topic that has been well-established for over a decade and is already included in major guidelines and society consensus such as those from the IDSA [8], the British Thoracic Society [9], and ESPID [10].

## Conclusions

The management of mild-moderate CAP in pediatric patients should prioritise evidence-based, targeted antibiotic therapy to ensure effective treatment while minimising the risk of AMR. To improve the management of CAP in pediatric patients, we have developed this consensus based on a thorough review of the best available evidence and extensive discussions with an expert panel. Amoxicillin remains the first-line therapy for bacterial CAP, with a recommended duration of 5 days for most cases. The decision to initiate antibiotics should be guided by clinical presentation, with careful consideration given to the viral etiology common in younger children. Considering regional variations in resistance patterns, high-dose amoxicillin (90 mg/kg/day) is still recommended in Italy, awaiting the results of other randomized controlled trials in Southern Europe, where a higher AMR rate is reported compared to Northern countries. For atypical pneumonia in older children, macrolides should be added if symptoms persist after 48 h of beta-lactam therapy. However, further efforts are needed. Future research should focus on enhancing diagnostic accuracy, optimizing antibiotic utilization, comparing the efficacy of different antibiotic regimens, and determining the optimal dosage and duration of treatment. Additionally, it is crucial to understand and address the barriers to implementing evidence-based guidelines in clinical practice.

By addressing these priorities, we can achieve more effective and responsible antibiotic use, ultimately improving outcomes for children with CAP.

## Electronic supplementary material

Below is the link to the electronic supplementary material.

Supplementary Material 1

Supplementary Material 2

## Data Availability

All the data are included in the manuscript.

## Abbreviations

CAP: Community Acquired Pneumonia
CRP: C-reactive Protein
ESPID: European Society For Paediatric Infectious Diseases
FIMMG: The Italian Federation of General Practitioners
FIMP: The Italian Federation of Paediatricians (FIMP)
GRADE: Grading of Recommendations Assessment, Development, and Evaluation
HICs: High-income countries
IDSA: Infectious Diseases Society of America
LMICs: Low- or middle-income countries
PCT: Procalcitonin
PRISMA: Preferred Reporting Items for Systematic Reviews and Meta-analyses
SIAIP: The Italian Society for Paediatric Allergy and Immunology
SIMG: The Italian Society of General Medicine and Primary Care
SIMRI: The Italian Society of Paediatric Respiratory Diseases,
SIP: The Italian Society for Paediatrics,
SIPPS: Italian Society for Preventive and Social Paediatrics
SITIP: The Italian Society of Pediatric Infectious Disease
RSV: Respiratory Syncytial Virus
WBC: White Blood Cell count
