# Supplementary material for: Treatment of mild to moderate community-acquired pneumonia in previously healthy children: an Italian intersociety consensus (SIPPS-SIP-SITIP-FIMP-SIAIP-SIMRI-FIMMG-SIMG)
Source: Ital J Pediatr. 2024 Oct 19;50:217. doi: 10.1186/s13052-024-01786-8 (PMC11491012; doi:10.1186/s13052-024-01786-8)
Supplement: Supplementary file 2 — Supplementary Material 2 [file 13052_2024_1786_MOESM2_ESM.docx]

**Supplementary Material**

**1. Protocol**

**2. Search Strategy**

**3. Excluded systematic reviews**

**4. Excluded observational studies or RCTs**

**5. Quality Assessment tool for Systematic review**

**6. Quality Assessment tool for Observational studies**

**6.1 Cohort studies**

**6.2 Cross-sectional studies**

**7. Quality Assessment tool for RCTs**

**8. Grading of Recommendations Assessment, Development, and Evaluation (GRADE).**

**Protocol**

**Document Structure**

This document aims to give the generalist paediatrician (family paediatrician, outpatient or hospital specialist) a practical and up-to-date tool. The document has been designed and structured according to three possible consultation methods:

**1. Summary of the recommendations:** The summary of recommendations contains only the final practical indications related to the chapters and questions

**2. Pathology sections**: the pathology sections deal with the topic *in extenso*. Each chapter includes two parts. First, an introductory narrative part, with updated summaries of the definition of the treated pathology, the clinical framework and the diagnostic management. The second part is developed according to the GRADE methodology related to antibiotic treatment in children with this pathology. We answered the questions based on research, analysis and synthesis of the scientific evidence and the "Evidence to the Decision" (EtD) process that explained and graded the recommendations

This document does not address issues related to children with comorbidities or underlying chronic disease, as specified below in the paragraph "Setting and reference population", management with therapies other than antibiotics and management of complications.

**3. Appendix:** Boxes, Figures and Tables included in the Appendix allow a quick consultation of the references, the adopted processes , the obtained results, and the recommendations formulation .

**Methods**

We decided to produce a Consensus Conference (CC) to provide for an analysis of the available evidence on the subject of the judicious use of antibiotic therapy in respiratory tract infections in developmental age. These issues may not have a complete sharing of opinions or conditions of uncertainty. This lack of generality can lead to inhomogeneity of behaviours on a clinical level, and the management aspects of assistance.

The Panel drew up the project and defined the general aims of the document and the specific objectives. We also established the time required for each phase and the coordination activity, the topics, the methods of consultation, the research and the selection of the studies, and the processes defining the Consensus by the Panel.

**Working Groups**

- Promoting Committee of the CC, which organized and directed the different stages of development of the CC;

- The Scientific and Technical Committee critically analyzed the literature, extracted and tabulated the relevant data, and elaborated the synthesis of the scientific literature. It also supported the formulation of recommendations according to the GRADE method. Finally, it prepared the questionnaire for voting on the recommendations according to the Delphi method and analyzed the results;

- Multidisciplinary and multi-professional panel or Jury panel (G), which elaborated the clinical questions, discussed the efficacy tests and formulated the recommendations, divided into subgroups by pathology in some work phases ;

- Writing group, which drafted the final text of the CC.

- A group of External Auditors of paediatricians and infectious disease specialists with expertise in the specific pathologies.

The Scientific Technical Committee and the Panel Committee held periodic meetings. The dates of the meetings and all preliminary versions of the document have been recorded.

The multidisciplinary and multi-professional panel includes paediatricians experts in infectious diseases, developmental age, general paediatricians and paediatricians of free choice. The panel also includes experts in allergology, clinical pharmacology, microbiology, epidemiology and research methodology and parent representatives.

The members of the Groups have been indicated by the Scientific Societies of the various disciplines or by the reference Associations.

We used the Delphi method to reach an agreement on the selected topics and the strength of the recommendations^^[[1]](#endnote-1)^^.

The external auditors did not participate in any phase of the development and drafting of the document, nor they voted for the recommendations.

**Consensus Conference Audience**

There is a wide group of primary users of the CC including family paediatricians, general paediatricians working in outpatient facilities and outpatient DEAs, paediatricians experts in infectious diseases, general practitioners (GPs) and Continuity of Care doctors.

The group also includes otolaryngology specialists, pulmonologists, immunologists, hygienists, pharmacologists, nurses and pharmacists involved in the management of children with respiratory infections. Parents and caregivers are also users of the document.

**Setting and target population**

This CC refers to the outpatient setting and emergency room management in the context of both Family Paediatrics and General Practitioners, or to hospitalised patients in the clinical conditions considered in this document, with the exclusions reported below. The CC provides recommendations on the management of children aged one month to 18 years with the following respiratory infectious diseases: pharyngotonsillitis (FT), acute and chronic sinusitis, acute otitis media (OMA) and recurrent acute otitis media (OMAR), community-acquired pneumonia (CAP). Children less than one month of age, with chronic diseases or comorbidities are excluded for the treatment of which we refer to the specific guidelines by pathology. The chronic diseases and comorbidities include primary or secondary cystic fibrosis and/or CFTR-pathies, primary ciliary dyskinesia, non-cystic fibrosis bronchiectasis, genetic diseases, known malformations of the cardio-respiratory system, neuromuscular diseases and other pre-existing chronic lung diseases, neoplasms, asthma, diabetes mellitus.

**Formulation of definitions and questions**

The definition of the treated pathology has been reported at the beginning of each chapter according to the available literature.

The questions and the outcomes shared and discussed within the panel have been identified. The questions were formulated by the methodology group using the "PICO" model (Patient/Population [P]; Intervention/Indicator [I]; Comparator/Control [C]; Outcome [O]) and developed according to the GRADE method.

The panel identified the outcomes a priori and then ranked(from 1 to 9) them in terms of importance in the decision-making process

Only outcomes categorized as “critical” or “important” were considered for the literature review and the formulation of the recommendation.

In particular, the outcomes related to antibiotic therapy of respiratory infections in children are the only ones of concern, while those related to diagnostic management and therapiescan be excluded. The treatment of the specific complications of the pathologies of concern have been neglected as well.

The subsets of the members' panel developed the clinical questions of the different pathologies.

**Research of Scientific Evidence**

We based the bibliographic search on the principle of hierarchical selection.

Firstly, summaries of evidence, Systematic Reviews, were sought.

We have also taken into account the most valid evidence-based LGs

The research was then completed, according to the principle of theoretical saturation, with the Primary Studies published after those included in the RS and with those considered relevant.

5.7.1. General Inclusion Criteria:

- Time limit of the search:

for RS: last ten years

for primary studies: from the date of closure of the bibliography of the RS included or, failing that, not older than ten years. We included the studies considered valid and relevant, found by manual research or indicated by experts regardless of the date of publication

- Publication language:

English, Italian. We also evaluated studies published in other languages if known to the authors, found by manual research or indicated by experts and considered valid and relevant.

- Population :

paediatric and adolescent patients, older than one month, without comorbidities or risk factors, suffering from the following respiratory infectious diseases: pharyngotonsillitis (FT), acute and chronic sinusitis, acute otitis media (OMA) and recurrent acute otitis media (OMAR), community-acquired pneumonia (CAP).

- Type of studies:

*Systematic Review, Meta-Analysis, Randomized Controlled Trial, Multicentre Study, Observational Study, Cohort Study, Longitudinal Study*

- Relevance to the clinical question

- Methodological validity, evaluated based on the minimum criteria described in the chapter "Analysis of scientific evidence".

The research strategy was discussed and agreed upon among the methodologists.

At least two authors carried out the research, evaluation of scientific evidence and data extraction. In case of disagreement, we decided after a discussion among the methodologists.

Research of Systematic Reviews and Primary Studies

**1. RS databases:** *Cochrane Library*, CDSR – *Cochrane Database of Systematic Reviews*, DARE – *Database of Abstract of Review of Effects In Cochrane Reviews*, *Other Reviews*, *Trials*

**2**. **PubMed** [*http:// www.ncbi.nlm.nih.gov/pubmed*](http://www.ncbi.nlm.nih.gov/pubmed)

**3. EMBASE** [*https://www.embase.com*](https://www.embase.com)

**4. SCOPUS** *https://www.scopus.com/*

**5. Manual Search**

**6. Bibliography from experts**

Keywords for Population, Intervention/Exposure Factor, Outcome and Search Strings for each question are reported in the Supplementary files.

**Analysis of scientific evidence**

We used validated checklists and criteria to perform the evidence analysis and evaluation.

We used the validated AGREE II tool^^[[2]](#endnote-2)^^]

For the evaluation of other consensus documents, we used the criteria defined by the SNLG^^[[3]](#endnote-3)^^

- Relevance of the topic
- Publication date < 3 years
- Multidisciplinary and multi-professional composition of the panel of experts
- Clear and detailed description of the methodology adopted and in line with the standards adopted by CNEC to assess the quality of scientific evidence

We used the validated tool AMSTAR 2 (*Assessment of Multiple Systematic Reviews*)^^[[4]](#endnote-4)^^.

Minimum score: overall judgement of high, moderate and low methodological quality.

We assessed any *bias* of the RCTs with the validated tool of the *Cochrane Collaboration* called "*Assessment of Risk of Bias*".^^[[5]](#endnote-5)^^

We used the Cochrane ROBINS-I tool for assessing the non-randomized controlled intervention trials, ^^[[6]](#endnote-6)^^

We used *Newcastle Ottawa Scales* to assess the observational studies: cohort, case-control, and cross-sectional*.*^^[[7]](#endnote-7)^^

We took the biases and the confounding factors into account to assess the quality of the studies.

Minimum validity criterion: absence of bias.

**GRADE Method**^^[[8]](#endnote-8)^,^[[9]](#endnote-9)^,^[[10]](#endnote-10)^^

**Grading the quality of the evidence**

| **Quality level** | **Meaning** | **Consequence** |
| --- | --- | --- |
| High | High degree of confidence in the results | It is unlikely that further studies will change confidence in effect estimation |
| Moderate | A fair degree of confidence in the results | Further studies may likely confirm or change confidence in effect estimation |
| Low | The results are hardly credible | More research is needed to obtain reliable estimates of the positive and negative effects of the intervention |
| Very low | The data examined is unreliable | You cannot rely on available effect estimates |

**Criteria for *upgrading* or *downgrading* the quality assessment (high, moderate, low, very low) of the tests**

| **Type of Tests**  **Randomized Controlled Trial = High**  **Observational study = low**  **Any other information = very low** | |
| --- | --- |
| **At.** Decrease  of the category  Attribution  (e.g. from "high" to  "moderate") | 1. Severe (-1 level) or very severe (-2 levels) limitations in the quality of study conduct  2. Inconsistency in results between different studies on the same question (-1 or -2 levels)  3. Some (-1 level) or important (-2 level) uncertainties about the direct transferability of results (*directness*)  4. Inaccuracy or insufficient data (*sparse data*) (-1 or -2 levels)  5. Possibility of selective publication and *reporting bias* (-1 or -2 levels) |
| **B.** Increase  of the category  Attribution  (e.g. from "low"  to "moderate") | 1. Strong intervention-outcome association, i.e. with relative risk >2 (<0.5), based on concordant evidence from two or more observational studies, without any plausible confounding factor (+1 level)  2. Very strong intervention-outcome association, i.e. with relative risk >5 (<0.2) (+2 levels)  3. Presence of a dose-response gradient (+1 level)  4. All possible confounding factors that could have altered the effect estimates would have reduced the observed effect (+1 level) |

**Outline of recommendations**

Recommendations Strength Assessments

Strong recommendation *for* action

Weak recommendation *in favour* of action

Weak recommendation *against* intervention

Strong Recommendation *Against* Intervention

**Determinants of the strength of the recommendation**

1) *Balance between desirable and undesirable effects*

If the difference between the magnitude of the desired and undesirable outcomes is large, the recommendation is strong (for or against the intervention). If the difference is small, the recommendation is weak.

2) *Overall quality of evidence for the outcomes considered*

The higher the quality of evidence for the outcomes considered, the stronger a recommendation.

3) *Values and preferences*

The more the assigned values and preferences diverge or the greater the uncertainty is, the greater the chance that the recommendation will be weak.

4) *Costs (resource allocation)*

The higher the costs of an intervention (i.e. the more resources consumed), the less likely it is to consider a strong recommendation.

Note. The formulation of the recommendations has been the subject of great attention and in-depth discussions, both on the substantive and formal aspects. Even in cases where good or moderate quality scientific evidence was not available, we noted that the authors tried to formulate shared recommendations, appropriate to the severity of the disease considered, taking into account the risks and benefits.That is, it is not unusual, nor wrong, to formulate strong recommendations based on low-quality evidence or even based on expert opinion.

**Approval of recommendations**

We used the Delphi method with a blinded questionnaire to vote on the recommendations.

We prepared five possible answers: *strongly agree, agree*, *neither agree nor disagree, disagree, strongly disagree.*

There are no unambiguous criteria for approving recommendations.

Informally, we considered that in several documents of good methodological quality, the panel approved the recommendations with a percentage of agreement equal to 70-75% *("strongly agree", "agree*").

We requested the reasons in case of *"neither agree nor disagree", "disagree", and "strongly disagree" answers*.

In any case, all the comments of the disagreeing votes were recorded and carefully considered, both on the content of the recommendation and on the formal correctness and clarity of exposition.

**GRADE-ADOLOPMENT**

The GRADE-ADOLOPMENT method is an evolution of the GRADE method that allows you to assess whether you can adapt to your context or adopt existing LG recommendations published to answer the PICO1 questions.

In this paper, we evaluated the possibility of adopting the recommendations of some LGs on the therapy of OMA and OMAR.

**Presentation, participation of Users and Users**

We presented a non-final version of this CC to the Jury of experts, nurses' and parents' associations. After in-depth discussion, the comments and collected observations were incorporated into the document when considered appropriate.

The Jury then defined the conclusions, and the writing committee drafted the preliminary consensus document.

The document was internally reviewed and approved by all panel members.

Four external reviewers assessed the document.

The authors approved the final draft in October 2023

**Software**

We used the RevMan 5.4.1 software^^[[11]](#footnote-1)^^, Nordic Cochrane Centre, The Cochrane Collaboration, 2014, to evaluate the methodological quality of the RCTs, the meta-analyses and related figures.

We used the GRADEpro GDT software, developed by the GRADE Working Group, for the overall quality of the evidence and the related tables.

**Update**

We will update the document after three years or in case of publication of new evidence that leads to changes to the recommendations.

**Implementation**

We will present the document at scientific meetings, and courses, as well as paediatric *forums and mailing lists*. We will give widespread information to family paediatricians, general paediatricians working in outpatient facilities and outpatient DEAs, paediatricians infectious disease specialists, general practitioners (GPs) and Continuity of Care doctors.

**Financing**

The SIPPS covered the costs for the document's drafting and publication (panel meetings, editing, printing and distribution). All the authors worked free of charge.

**Conflict of interest**

Each of the members of the working groups signed a declaration on possible conflicts of interest (CI) in the preliminary stages of the project and at the end of the project.

Management of any CIs:

- the members of the methodology team and the external auditors did not have CI;
- the authors with any ICs did not participate, therefore, in the systematic review of the evidence, but participated in all the other phases of implementations, contributing as according to their competence;
- the methodology group and the authors without ICs checked the correctness and consistency of each part of the document and, in particular, of the recommendations;
- each author could vote, express and justify any disagreement anonymously;
- we discussed the results of the votes and the reasons for any disagreements collectively towards the final version of the conclusions and recommendations.

**References**

Boulkedid R, Abdoul H, Loustau M, et al. Using and Reporting the Delphi Method for Selecting Healthcare Quality Indicators: A Systematic Review. PLoS One. 2011;6:e20476

Brouwers M, Kho ME, Browman GP, et al. for the AGREE Next Steps Consortium. AGREE II: Advancing guideline development, reporting and evaluation in healthcare. Can Med Assoc J. 2010. Available online July 5, 2010

SNLG. Good clinical-care practices. Available in <https://snlg.iss.it/?cat=4> (last accessed 24-07-2021)

Shea BJ, Reeves BC, Wells G, et al. AMSTAR 2: a critical appraisal tool for systematic reviews that include randomised or non-randomised studies of healthcare interventions, or both. BMJ. 2017; 358:J4008

Higgins, J.P.T.; Thomas, J.; Chandler, J.; Cumpston, M.; Li, T.; Page, M.J.; Welch, V.A. (Eds.). Cochrane Handbook for Systematic Reviews of Interventions Version 6.2 (Updated February 2021); Cochrane: 2021. Available online: www.training.cochrane.org/handbook (accessed on 15 July 2022).

Sterne, J.A.C.; Hernán, M.A.; Reeves, B.C.; Savović, J.; Berkman, N.D.; Viswanathan, M.; Henry, D.; Altman, D.G.; Ansari, M.T.; Boutron, I.; et al. ROBINS-I: A tool for assessing risk of bias in non-randomized studies of interventions. BMJ 2016, 355, i4919e

Wells GA, Shea B, O'Connell D, et al. The Newcastle-Ottawa Scale (NOS) for assessing the quality of nonrandomized studies in meta-analyses, 2012. Available at: <http://wwwohrica/programs/clinical_epidemiology/oxfordasp> (last accessed 24-07-2021)

Schünemann HJ, Oxman AD, Brozek J, et al. GRADE Working Group. Grading quality of evidence and strength of recommendations for diagnostic tests and strategies. BMJ. 2008; 336:1106-10

Guyatt GH, Oxman AD, Kunz R, et al. GRADE Working Group. Going from evidence to recommendations. BMJ. 2008;336:1049-51

Guyatt GH, Oxman AD, Kunz R, et al. GRADE working group. Incorporating considerations of resource use into grading recommendations. BMJ. 2008;336:1170-73

**2. Search Strategy**

(newborn* OR neonat* OR infan* OR toddler* OR pre-schooler* OR preschooler* OR child* OR children OR adolescen* OR pediatr* OR paediatr* OR youth* OR teen* OR kid OR baby OR babies)

**AND**

("community-acquired pneumon*" OR "community acquired pneumon*" OR "community-acquired bronchopneumon*" OR "community acquired bronchopneumon*" OR "CAP" OR "C.A.P." OR “pleural effusion” OR “round pneumonia” OR “interstitial pneumon*” OR “atypical pneumon*”)

**AND**

("anti-bacterial*" OR "antibacterial*" OR "antibiotic*" OR "anti-infective" OR "bactericid*" OR "bacteriocid*" OR "antimicrobial*" OR "treatment*" OR "therap*")

**AND**

(("first-line therap*" OR "first line therap*" OR “first line management*” OR “first-line management” OR “first line treatment” OR “first-line treatment”) OR ("second-line therap*" OR "second line therap*" OR “second line management*” OR “second-line management” OR “second-line treatment” OR “second line treatment”) OR ("posology" OR "dose" OR "dosage" OR "duration of administration" OR "time of administration" OR “days of therapy” OR “days of treatment” OR “DOT” OR “length of therapy” OR “length of treatment” OR “LOT” OR "method of administration" OR “route of administration” OR "intravenous" OR "intramuscular" OR “oral” OR “systemic therap*” OR “systemic treatment”) OR ("clinical healing" OR "healing" OR "clinical recovery" OR "recovery" OR "clinical improvement*" OR "clinical worsening" OR "worsening" OR "clinical deterioration" OR "clinical decay" OR “clinical outcome*” OR “outcome*”) OR ("allergy to penicillin*" OR "penicillin allergy" OR “penicillin hypersensitivity” OR “penicillin side effect*” OR “beta-lactam allergy” OR “beta-lactam hypersensitivity”) OR ("Drug-Related Side Effects and Adverse Reactions" OR "adverse effect*" OR "adverse drug reaction*" OR "drug side effect*") OR ("drug resistan*" OR "antimicrobial resistan*" OR “antibacterial resistan*” OR “antibiotic resistan*”))

**AND**

Limit: January 2012 – April 2024

**3. Excluded systematic reviews**

| **Title** | **Reason for exclusion** |
| --- | --- |
| Antibiotic use for community-acquired pneumonia in neonates and children: WHO evidence review  (2018, Mathur) | Wrong population (Hospitalised children with severe and very severe pneumonia) |
| Evidence for short duration of antibiotic treatment for non-severe community acquired pneumonia (CAP) in children - are we there yet? A systematic review of randomised controlled trials  (2014, Ben Shimol) | Only one study from high income country |
| Short-course versus long-course oral antibiotic treatment for infections treated in outpatient settings: a review of systematic reviews (Dawson-Hahn, 2017) | The studies about pneumoniae are conducted in lower-middle income countries or in adult populations |
| Clinical cure rates in subjects treated with azithromycin for community-acquired respiratory tract infections caused by azithromycin-susceptible or azithromycin-resistant Streptococcus pneumoniae: analysis of Phase 3 clinical trial data (Zhanel, 2014) | Separate data for children not available |
| Evaluation of the efficacy of erythromycin compared to other macrolides in adults or adolescents with community-acquired pneumonia (CAP): Systematic review and meta-analysis of randomized controlled clinical trials (Ashi, 2020) | Conference abstract |
| Delayed antibiotic prescribing for respiratory tract infections: individual patient data meta-analysis (Stuart, 2021) | Wrong outcome |
| Systematic review and meta-analysis of the safety of erythromycin compared to clarithromycin in adults and adolescents with pneumonia (Eljaaly et al., 2020) | Data on children not available |
| Short-course versus long-course therapy of the same antibiotic for community-acquired pneumonia in adolescent and adult outpatients (López-Alcalde et al., 2018) | No study included in the review |
| Efficacy of tetracyclines and fluoroquinolones for the treatment of macrolide-refractory Mycoplasma pneumoniae pneumonia in children: a systematic review and meta-analysis (Ahn, 2021) | Wrong population |
| Efficacy of erythromycin compared to clarithromycin and azithromycin in adults or adolescents with community-acquired pneumonia: A Systematic Review and meta-analysis of randomized controlled trials. (Ashy N, 2022) | Wrong population |
| Developing Consensus on Clinical Outcomes for Children with Mild Pneumonia: A Delphi Study. (Florin TA, 2023) | Wrong study design |
| Childhood community-acquired pneumonia. (Meyer Sauteur PM, 2024) | Wrong study design |

**4. Excluded observational studies or RCTs**

|  | |
| --- | --- |
| **Title** | **Reason** |
| The Association of Antibiotic Duration with Successful Treatment of Community-Acquired Pneumonia in Children (Same, 2021) | Wrong population (inpatient, complicated CAP) |
| Changing clinical practice: management of paediatric community-acquired pneumonia  (Elemraid, 2014) | Wrong outcome |
| Use of antibiotics and factors associated with treatment failure among 152,245 patients with pneumonia treated in the community — a retrospective cohort study (Reiner-Benaim, 2022 ) | Not separate paediatric data |
| Randomized double-blind controlled trial of short vs. Standard course outpatient therapy of community acquired pneumonia in children (SCOUT-CAP) (Williams, 2020) | Conference abstract |
| Short-course antimicrobial therapy for paediatric respiratory infections (SAFER): A multicentre, randomized, controlled, blinded, noninferiority trial (Pernica, 2020) | Conference abstract |
| Short-course antimicrobial therapy for paediatric respiratory infections (SAFER): study protocol for a randomized controlled trial (Pernica, 2018) | Study protocol |
| Evolution of acute infection with atypical bacteria in a prospective cohort of children with community-acquired pneumonia receiving amoxicillin (Nascimento-Carvalho, 2017) | Low-income countries |
| Comparison of oral amoxicillin given thrice or twice daily to children between 2 and 59 months old with non-severe pneumonia: A randomized controlled trial (Vilas-Boas, 2014) | Low-income countries |
| Clinical Profile And Comparison Of Narrow Versus Broad Spectrum Antibiotic Therapy For Community Acquired Pneumonia In Children Hospitalized At Tertiary Care Centre (Sharma, 2020) | Low-income countries |
| Efficacy and safety of tosufloxacin tosilate hydrate for the treatment of community-acquired pneumonia in children (Sakata, 2014) | No full text available |
| Comparative Effectiveness of Empiric b-Lactam Monotherapy and b-Lactam–Macrolide Combination Therapy in Children Hospitalized with Community-Acquired Pneumonia (Ambroggio 2012) | Wrong population (inpatient, complicated CAP) |
| Effectiveness of β-Lactam Monotherapy  vs Macrolide Combination Therapy for Children Hospitalized With Pneumonia (Williams, 2017) | Wrong population (inpatient, complicated CAP) |
| Comparative Effectiveness of Ceftriaxone in Combination with a Macrolide Compared with Ceftriaxone Alone for Pediatric Patients Hospitalized with Community Acquired Pneumonia (Leyenaar, 2014) | Wrong population (inpatient, complicated or severe CAP) |
| Rapid defervescence after doxycycline treatment of macrolide-resistant Mycoplasma pneumoniae–associated community-acquired pneumonia in children (Lung, 2013) | Wrong population (inpatient, complicated CAP) |
| Antibiotic Use and Outcomes in Young Children  Hospitalized With Uncomplicated Community-Acquired Pneumonia (Hofto, 2022) | Wrong outcome |
| IV Penicillin G is as effective as IV cefuroxime in treating community-acquired pneumonia in children (Amarilyo, 2014) | Wrong population (inpatient, complicated CAP) |
| Pediatric community-acquired pneumonia treated with a three-day course of tebipenem pivoxil (Sakata, 2017) | Wrong study design |
| Amoxicillin and Penicillin G Dosing in Pediatric Community-Acquired Pneumococcal Pneumonia in the Era of Conjugate Pneumococcal Vaccines (Huynh D., 2022) | Wrong study design |
| Community-acquired bacterial pneumonia in children: an update on antibiotic duration and immunization strategies (Lyon, E., 2024) | Wrong study design |
| Discharge Antibiotic Prescribing at Children's Hospitals with Established Antimicrobial Stewardship Programs (Same R., 2022) | Wrong outcome |
| Adherence to Short-Term Antibiotic Therapy in Children - a Blinded, Prospective, Electronically-Monitored Study (Youngster I., 2022) | Wrong outcome |
| Clinical characteristics of hospitalized children with community-acquired pneumonia and respiratory infections: Using machine learning approaches to support pathogen prediction at admission (Chang, T.-H., 2023) | Wrong outcome |
| Clinical Features and Associated Factors of Macrolide-Unresponsive Mycoplasma pneumonia and Efficacy Comparison Between Doxycycline, Tosufloxacin and Corticostreoid as a Second-Line Treatment (Kang et al, 2023) | Wrong outcome |
| Extended Versus Standard Antibiotic Course Duration in Children <5 Years of Age Hospitalized With Community-acquired Pneumonia in High-risk Settings: Four-week Outcomes of a Multicenter, Double-blind, Parallel, Superiority Randomized Controlled Trial. (McCallum GB, 2022) | Wrong countries |
| Antibiotic use and outcomes among children hospitalized with suspected pneumonia. | Wrong comparison |
| Amoxicillin for childhood pneumonia: 3 days versus 7 days. (2021) | Wrong publication type |
| Variation in bacterial pneumonia diagnoses and outcomes among children hospitalized with lower respiratory tract infections. (Cotter JM, 2022) | Wrong outcome |
| Comparable Outcomes With Five and 10 Days of Antibiotics in Children With CAP. (Barry HC, 2022) | Wrong publication type |
| Evaluation of a 5-day High-Dose Course of Amoxicillin for the Management of Community-Acquired Pneumonia in Children of 6 months to 10 years of Age. (Joerger T, 2022) | Wrong publication type |
| Improving Short Course Treatment of Pediatric Infections: A Randomized Quality Improvement Trial (Vernacchio, 2024) | Wrong outcome |
| How Long Antibiotic Treatment Is Needed for Community-acquired Pneumonia in Children? (Kuitunen, 2024) | Wrong publication type |
| Amoxicillin Dose and Duration of Treatment and Need for Antibiotic Re-treatment in Children with Community-Acquired Pneumonia (Park, B.L., 2022) | Wrong publication type |
| Optimizing Antibiotic Durations of Therapy in Pediatric Community-acquired Pneumonia (Cook G., 2023) | Conference abstract |

**5. Quality Assessment tool of Systematic review**

| **AMSTAR 2** | **Antibiotic Treatment Duration for Community-Acquired Pneumonia in Outpatient Children in High-Income Countries—A Systematic Review and Meta-Analysis**  **Kuitunen et al., 2022 (32)** | **Short-Course vs Long-Course Antibiotic Therapy for Children**  **With Nonsevere Community-Acquired Pneumonia**  **A Systematic Review and Meta-analysis**  **Qinyuan Li, 2022 (33)** | **Shorter Versus Longer-term Antibiotic Treatments for Community-Acquired Pneumonia in Children: A Meta-analysis**  **Ya Gao, 2023 (34)** | **Shorter versus longer duration of Amoxicillin‑based treatment for pediatric patients with community‑acquired pneumonia: a systematic review and meta‑analysis**  **Isabela R. Marques et al., 2022 (35)** | **Efficacy of erythromycin compared to clarithromycin and azithromycin in adults or adolescents with community-acquired pneumonia: A Systematic Review and meta-analysis of randomized controlled trials.**  **Ashy et al., 2022 (37)** | **Treatment of mycoplasma pneumonia: A systematic review**  **Biondi et al., 2014 (29)** | **Antibiotics for community-acquired pneumonia in children**  **Lodha et al., 2013 (31)** | **Comparative efficacy of beta-lactams and macrolides in the treatment of pediatric pneumonia: A systematic review**  **Saeedy, 2020 (36)** | **Antibiotics for community-acquired lower respiratory tract infections**  **secondary to Mycoplasma pneumoniae in children**  **Gardiner, 2015 (30)** |
| --- | --- | --- | --- | --- | --- | --- | --- | --- | --- |
| **1. Did the research questions and inclusion criteria for the review include the components of PICO?** | Yes | Yes | Yes | Yes | Yes | Yes | Yes | Yes | Si Yes |
| **2. Did the report of the review contain an explicit statement that the review methods were established prior to the conduct of the review and did the report justify any significant deviations from the protocol?** | Yes | Yes | Yes | Yes | Partial Yes (no motivation for deviation from the protocol) | Yes | Yes | Yes | Yes |
| **3. Did the review authors explain their selection of the study designs for inclusion in the review?** | Yes | Yes | Yes | Yes | Yes | Yes | Yes | Yes | Yes |
| **4. Did the review authors use a comprehensive literature search strategy?** | Partial Yes (3 database) | Yes (4 international database + 3 chinise + references + clinicaltrials + WHO) | Yes (4 database) | Partial Yes (3 database) | Partial Yes (3 database + clinicatrials but not references) | Yes | Partial Yes (No Pubmed) | Yes | Yes |
| **5. Did the review authors perform study selection in duplicate?** | Yes | Yes | Yes | Yes | Yes | Yes | Yes | Yes | Yes |
| **6. Did the review authors perform data extraction in duplicate?** | Yes | Yes | Yes | Yes | Yes | Yes | Yes | Yes | Yes |
| **7.** **Did the review authors provide a list of excluded studies and justify the exclusions?** | Partial yes (motivation for esclusion but not list of escluded studies) | Yes | No | Partial yes (list of excluded studies without motivation) | No | No | Yes | No | Yes |
| **8. Did the review authors describe the included studies in adequate detail?** | Yes | Yes | Yes | Yes | Partial yes (no description of population included) | Yes | Yes | Yes | Yes |
| **9. Did the review authors use a satisfactory technique for assessing the risk of bias (RoB) in individual studies that were included in the review?** | Yes | Yes | Yes | Yes | Yes | Yes | Yes | Yes | Yes |
| **10. Did the review authors report on the sources of funding for the studies included in the review?** | No | No | No | No | Yes | Yes | No | No | Yes |
| **11. If meta-analysis was performed did the review authors use appropriate methods for statistical combination of results?** | Yes | Yes | Yes | Yes | Yes | Yes | No meta-analysis | Not reported in materials and methods | No meta-analysis |
| **12. If meta-analysis was performed, did the review authors assess the potential impact of RoB in individual studies on the results of the meta-analysis or other evidence synthesis?** | Yes | Yes | Yes | Yes | No | Yes | No meta-analysis | No | No meta-analysis |
| **13. Did the review authors account for RoB in individual studies when interpreting/ discussing the results of the review?** | Yes | Yes | Yes | Yes | No | Yes | Yes | No | Yes |
| **14. Did the review authors provide a satisfactory explanation for, and discussion of, any heterogeneity observed in the results of the review?** | Yes | Yes | Yes | Yes | Yes | Yes | Yes | Yes | Yes |
| **15. If they performed quantitative synthesis did the review authors carry out an adequate investigation of publication bias (small study bias) and discuss its likely impact on the results of the review?** | Yes | Yes | Yes | Yes | Yes | Yes | No meta-analysis | Yes | No meta-analysis |
| **16. Did the review authors report any potential sources of conflict of interest, including any funding they received for conducting the review?** | Yes | Yes | Yes | Yes | Yes | Yes | Yes | Yes | Yes |
| TOTAL ASSESSMENT | Moderate | Moderate | Moderate | Moderate | Very Low | Moderate | Moderate | Very Low | Moderate |
|  | 1 partial critical  1 not critical | 1 not critical | 1 critical e 1 partial critical | 1 partial critical,  1 not critical | 3 critical  2 not critical | 1 partial critical  0 not critical | 1 not critical | 2 critical  1 partial critical  2 not critical | No meta-alysis |

**6. Quality Assessment tool of Observational studies**

**6.1 Cohort studies**

|  | **Newcastle Quality Assessment Scale COHORT STUDIES** |  |  |  |  |  |  |  |  |  |
| --- | --- | --- | --- | --- | --- | --- | --- | --- | --- | --- |
|  | **Selection** |  |  |  | **Comparability** | **Outcome** |  |  |  |  |
|  | **Representativeness of the exposed cohort** | **Selection of the non exposed cohort** | **Ascertainment of exposure** | **Demonstration that outcome of interest was not present at start of study** | **Comparability of cohorts on the basis of the design or analysis** | **Assessment of outcome** | **Was follow-up long enough for outcomes to occur** | **Adequacy of follow up of cohorts** | **Total** | **Assessment** |
| **Short- Versus Prolonged-Duration Antibiotics for Outpatient Pneumonia in Children**  **Shapiro, 2021 (47)** |  |  |  | * | * | ** | * | * | 6 | moderate |
| **Antibiotic Choice and Clinical Outcomes in Ambulatory Children with Community- Acquired Pneumonia**  **Lipsett, 2021 (46)** |  | * |  | * | * | * | * | * | 6 | moderate |
| **Comparative Effectiveness of Beta-lactam vs. Macrolide monotherapy in Children with Pneumonia Diagnosed in the Outpatient Setting**  **Ambroggio, 2015 (42)** |  | * | * | * | * | * | * | * | 7 | good |
| **Beta-Lactam Versus Beta-Lactam/Macrolide Therapy in Pediatric Outpatient Pneumonia**  **Ambroggio, 2016 (43)** |  | * | * | * | * | * | * | * | 7 | good |
| **Antibiotic Treatment for Children Hospitalized With Community-Acquired Pneumonia After Oral Therapy**  **Breuer et al, 2014 (55)** |  | * | * | * | * | * |  |  | 5 | moderate |
| **Antibiotic Treatment of Children With Community-Acquired Pneumonia: Comparison of Penicillin or Ampicillin Versus Cefuroxime**  **Dinur-Schejter, 2012 (54)** |  | * | * | * | * | * |  |  | 5 | moderate |
| **Comparative Effectiveness of Empiric Antibiotics for Community-Acquired Pneumonia**  **Queen, 2014 (49)** |  | * | * | * | * | * |  | * | 6 | moderate |
| **Narrow Vs Broad-spectrum Antimicrobial Therapy for Children Hospitalized With Pneumonia**    **Williams, 2013 (51)** |  | * | * | * | * | * | * | * | 7 | good |
| **Effectiveness of β-Lactam Monotherapy**  **vs Macrolide Combination Therapy for Children**  **Hospitalized With Pneumonia**  **Williams et al., 2017 (52)** |  | * | * | * | * | * | * |  | 6 | moderate |
| **Comparative Effectiveness of Empiric b-Lactam Monotherapy**  **and b-Lactam–Macrolide Combination Therapy in Children**  **Hospitalized with Community-Acquired Pneumonia**  **Ambroggio et al., 2012 (43)** |  | * | * | * | * | * | * | * | 7 | good |

**6.2 Cross sectional studies**

|  | **Newcastle Quality Assessment Scale CROSS-SECTIONAL STUDIES** |  |  |  |  |  |  |  |  |
| --- | --- | --- | --- | --- | --- | --- | --- | --- | --- |
|  | **Selection** |  |  |  | **Comparability** | **Outcome** |  |  |  |
|  | **Representativeness of the sample** | **Sample size** | **Non-respondents** | **Ascertainment of the exposure (risk factor)** | **The subjects in different outcome groups are comparable, based on the study design**  **or analysis. Confounding factors are controlled** | **Assessment of the outcome** | **Statistical test** | **Total** | **Assessment** |
| **Effects of clinical pathway implementation on antibiotic prescriptions for pediatric community-acquired pneumonia**  **Donà et al., 2018 (45)** |  |  | * | * | * | ** | * | 6 | moderate |
| **Impact of a Guideline on Management of Children Hospitalized With Community-Acquired Pneumonia**  **Newman, 2012 (50)** |  |  | * | * | * | ** | * | 6 | moderate |
| **Management of Pediatric Pneumonia: A Decade After the Pediatric Infectious Diseases Society and Infectious Diseases Society of America Guideline**  **Ambroggio et al, 2023 (48)** |  |  | * | * | * | ** | * | 6 | moderate |
| **Evaluation of a Pediatric Community-Acquired Pneumonia**  **Antimicrobial Stewardship Intervention at an Academic**  **Medical Center**  **Puzz et al., 2023 (53)** |  |  | * | * | * | ** | * | 6 | moderate |

**7. Quality Assessment tool for RCTs**

**RCT (Nordic Cochrane Centre, The Cochrane Collaboration. Review Manager 5 (RevMan 5). Version 5.3. Copenhagen: Nordic Cochrane Centre, The Cochrane Collaboration, 2014)**

**Risk of bias summary: review authors' judgements about each risk of bias item for each included study.**

**Risk of bias graph: review authors' judgements about each risk of bias item presented as percentages across all included studies**


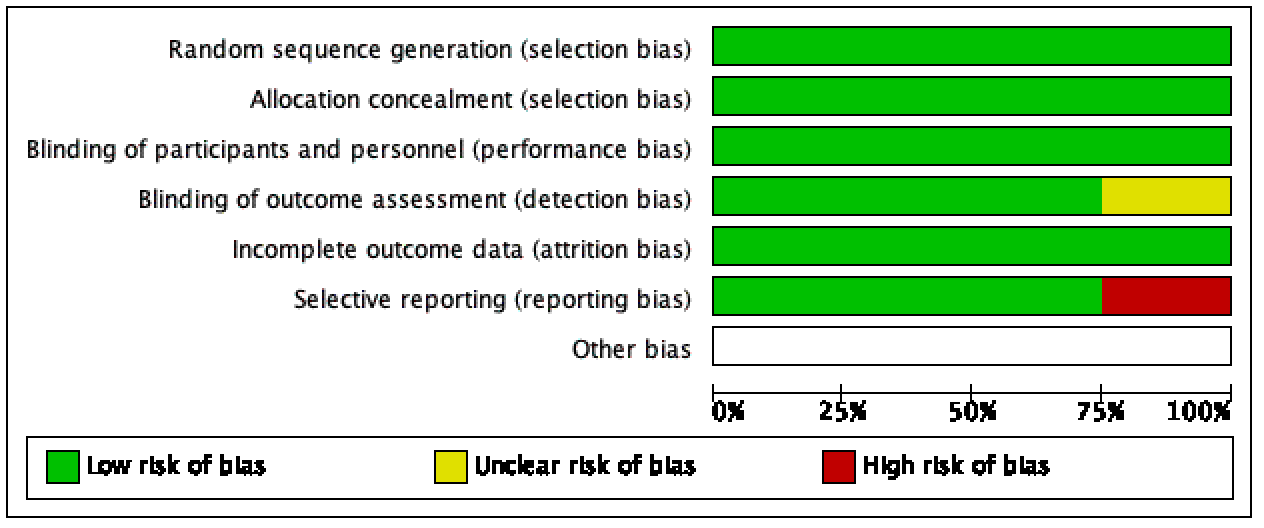


**8. Grading of Recommendations Assessment, Development, and Evaluation (GRADE).**

**Supplementary Material 2**

**QUESTION 1 - What is the first-line antibiotic for treating mild-to-moderate CAP in a child under five years old with a complete immunization schedule (at least two doses of hexavalent and pneumococcal vaccines)?**

***Amoxicillin compared to broad-spectrum antibiotics for CAP in children with a complete immunization schedule***

| **Certainty assessment** | | | | | | | **№ of patients** | | **Effect** | | **Certainty** | **Importance** |
| --- | --- | --- | --- | --- | --- | --- | --- | --- | --- | --- | --- | --- |
| **№ of studies** | **Study design** | **Risk of bias** | **Inconsistency** | **Indirectness** | **Imprecision** | **Other considerations** | **Amoxicillina** | **broad-spectrum antibiotics** | **Relative (95% CI)** | **Absolute (95% CI)** |  |  |
| **Treatment failure (assessed with: New antibiotic prescription, follow-up 14 days)** | | | | | | | | | | | | |
| 1^46^ | non-randomised studies | serious^a^ | serious^b^ | not serious | not serious | none | 2909/65872 (4.4%) | 3204/62381 (5.1%) | **OR 0.85**  (0.81 to 0.90) | **7 fewer per 1.000** (from 9 fewer to 5 fewer) | ⨁◯◯◯ Very low | IMPORTANT |
| **Treatment failure (assessed with: need of hospitalization/re-hospitalization, follow-up 14 days)** | | | | | | | | | | | | |
| 1^46^ | non-randomised studies | serious^a^ | serious^b^ | not serious | not serious | none | 428/65872 (0.6%) | 526/62381 (0.8%) | **OR 0.77** (0.68 to 0.87) | **2 fewer per 1.000** (from 3 fewer to 1 fewer) | ⨁◯◯◯ Very low | IMPORTANT |
| **Treatment outcome (assessed with: progression to severe or complicated CAP or admission to ICU, follow-up 14 days)** | | | | | | | | | | | | |
| 1^46^ | non-randomised studies | serious^a^ | serious^b^ | not serious | not serious | none | 35/65872 (0.1%) | 40/62381 (0.1%) | **OR 0.83** (0.53 to 1.30) | **0 fewer per 1.000** (from 0 fewer to 0 fewer) | ⨁◯◯◯ Very low | IMPORTANT |

**CI:** confidence interval; **OR:** odds ratio

#### Explanations

a. moderate quality of study

b. diagnostic uncertainty

References

46. Lipsett, 2021

***Amoxicillin compared to Macrolides for CAP in children with a complete immunization schedule***

| **Certainty assessment** | | | | | | | **№ of patients** | | **Effect** | | **Certainty** | **Importance** |
| --- | --- | --- | --- | --- | --- | --- | --- | --- | --- | --- | --- | --- |
| **№ of studies** | **Study design** | **Risk of bias** | **Inconsistency** | **Indirectness** | **Imprecision** | **Other considerations** | **Amoxicillin** | **Macrolides** | **Relative (95% CI)** | **Absolute (95% CI)** |  |  |
| **Treatment failure (assessed with: New antibiotic prescription, follow-up 14 days)** | | | | | | | | | | | | |
| 1^46^ | non-randomised studies | serious^a^ | serious^b^ | not serious | not serious | none | 2909/65872 (4.4%) | 4697/108917 (4.3%) | **OR 1.03** (0.98 to 1.07) | **1 more per 1.000** (from 1 fewer to 3 more) | ⨁◯◯◯ Very low | IMPORTANT |
| **Treatment failure (assessed with: Need of hospitalization/re-hospitalization, follow-up 14 days)** | | | | | | | | | | | | |
| 1^46^ | non-randomised studies | serious^a^ | serious^b^ | not serious | not serious | none | 428/65872 (0.6%) | 402/108917 (0.4%) | **OR 1.77** (1.54 to 2.02) | **3 more per 1.000** (from 2 more to 4 more) | ⨁◯◯◯ Very low | IMPORTANT |
| **Treatment failure (assessed with: Progression to severe/complicated CAP or admission to ICU, follow-up 14 days)** | | | | | | | | | | | | |
| 1^46^ | non-randomised studies | serious^a^ | serious^b^ | not serious | not serious | none | 35/65872 (0.1%) | 28/108917 (0.0%) | **OR 2.07** (1.26 to 3.40) | **0 fewer per 1.000** (from 0 fewer to 1 more) | ⨁◯◯◯ Very low | IMPORTANT |

**CI:** confidence interval; **OR:** odds ratio

#### Explanations

a. moderate quality of study

b. diagnostic uncertainty

References

46. Lipsett, 2021

***Beta-lactams compared to Macrolides for CAP in children with a complete immunization schedule***

| **Certainty assessment** | | | | | | | **№ of patients** | | **Effect** | | **Certainty** | **Importance** |
| --- | --- | --- | --- | --- | --- | --- | --- | --- | --- | --- | --- | --- |
| **№ of studies** | **Study design** | **Risk of bias** | **Inconsistency** | **Indirectness** | **Imprecision** | **Other considerations** | **betalactams** | **Macrolides** | **Relative (95% CI)** | **Absolute (95% CI)** |  |  |
| **Treatment failure (assessed with: clinical cure, follow-up 7 days)** | | | | | | | | | | | | |
| 5^a,29^ | randomised trials | serious^b^ | not serious | not serious | not serious | publication bias strongly suspected^c^ | 178/255 (69.8%) | 164/190 (86.3%) | **OR 0.37** (0.22 to 0.60) | **163 fewer per 1.000** (from 282 fewer to 72 fewer) | ⨁⨁◯◯ Low | IMPORTANT |

**CI:** confidence interval; **OR:** odds ratio

#### Explanations

a. 5 RCTs included in a meta-analysis

b. most of the studies included in the systematic review have bias (performance, detection, attrition) or conflict of interest

c. publication bias in small studies regarding efficacy of treatment

References

29. Biondi, 2014

***Beta-lactams compared to beta-lactams + Macrolides for CAP in children with a complete immunization schedule***

| **Certainty assessment** | | | | | | | **№ of patients** | | **Effect** | | **Certainty** | **Importance** |
| --- | --- | --- | --- | --- | --- | --- | --- | --- | --- | --- | --- | --- |
| **№ of studies** | **Study design** | **Risk of bias** | **Inconsistency** | **Indirectness** | **Imprecision** | **Other considerations** | **betalactams** | **betalactams + macrolides** | **Relative (95% CI)** | **Absolute (95% CI)** |  |  |
| **Treatment failure (assessed with: new prescription of antibiotic, follow-up 14 days)** | | | | | | | | | | | | |
| 1^46^ | non-randomised studies | serious^a^ | serious^b^ | not serious | not serious | none | 6113/128253 (4.8%) | 273/11719 (2.3%) | **OR 2.10** (1.86 to 2.37) | **24 more per 1.000** (from 19 more to 30 more) | ⨁◯◯◯ Very low | IMPORTANT |
| **Treatment failure (assessed with: Need of hospital admission/re-hospital admission, follow-up 14 days)** | | | | | | | | | | | | |
| 1^46^ | non-randomised studies | serious^a^ | serious^b^ | not serious | not serious | none | 954/128253 (0.7%) | 87/11719 (0.7%) | **OR 1.00** (0.80 to 1.25) | **0 fewer per 1.000** (from 1 fewer to 2 more) | ⨁◯◯◯ Very low | IMPORTANT |
| **Treatment failure (assessed with: Progression to severe/complicated pneumonia or admission to PICU, follow-up 14 days)** | | | | | | | | | | | | |
| 1^46^ | non-randomised studies | serious^a^ | serious^b^ | not serious | not serious | none | 75/128253 (0.1%) | 7/11719 (0.1%) | **OR 0.98** (0.45 to 2.12) | **0 fewer per 1.000** (from 0 fewer to 1 more) | ⨁◯◯◯ Very low | IMPORTANT |
| **Treatment failure (assessed with: Need of hospital admission/re-admission, follow-up 3-10 weeks)** | | | | | | | | | | | | |
| 1^52^ | non-randomised studies | serious^a^ | not serious | not serious | not serious | none | 33/616 (5.4%) | 5/257 (1.9%) | **OR 2.85** (1.10 to 7.39) | **34 more per 1.000** (from 2 more to 108 more) | ⨁◯◯◯ Very low | IMPORTANT |
| **Treatment failure (assessed with: Progression to severe/complicated pneumonia or admission to PICU, follow-up 3-10 weeks)** | | | | | | | | | | | | |
| 1^52^ | non-randomised studies | serious^a^ | not serious | not serious | not serious | none | 57/1017 (5.6%) | 25/399 (6.3%) | **OR 0.89** (0.55 to 1.44) | **7 fewer per 1.000** (from 27 fewer to 25 more) | ⨁◯◯◯ Very low | IMPORTANT |

**CI:** confidence interval; **OR:** odds ratio

#### Explanations

a. moderate quality of study

b. diagnostic uncertainty

References

46. Lipsett, 2021

52. Williams, 2017

***Amoxicillin compared to Macrolides for CAP in children younger than 5 years of age with a complete immunization schedule***

| **Certainty assessment** | | | | | | | **№ of patients** | | **Effect** | | **Certainty** | **Importance** |
| --- | --- | --- | --- | --- | --- | --- | --- | --- | --- | --- | --- | --- |
| **№ of studies** | **Study design** | **Risk of bias** | **Inconsistency** | **Indirectness** | **Imprecision** | **Other considerations** | **amoxicillin** | **macrolides** | **Relative (95% CI)** | **Absolute (95% CI)** |  |  |
| **Treatment failure (assessed with: New prescription of antibiotics,** **follow-up 14 days)** | | | | | | | | | | | | |
| 1^42^ | non-randomised studies | not serious | serious^a^ | not serious | not serious | none | 10/339 (2.9%) | 9/339 (2.7%) | **OR 1.11** (0.45 to 2.78) | **3 more per 1.000** (from 14 fewer to 44 more) | ⨁◯◯◯ Very low | IMPORTANT |

**CI:** confidence interval; **OR:** odds ratio

#### Explanations

a. diagnostic uncertainty

References

42. Ambroggio, 2015

***Beta-lactams compared to beta-lactams + Macrolides for CAP in children younger than 5 years of age with a complete immunization schedule***

| **Certainty assessment** | | | | | | | **№ of patients** | | **Effect** | | **Certainty** | **Importance** |
| --- | --- | --- | --- | --- | --- | --- | --- | --- | --- | --- | --- | --- |
| **№ of studies** | **Study design** | **Risk of bias** | **Inconsistency** | **Indirectness** | **Imprecision** | **Other considerations** | **betalactams** | **betalactams + macrolides** | **Relative (95% CI)** | **Absolute (95% CI)** |  |  |
| **Treatment failure (assessed with: new prescription of antibiotics,** **follow-up 14 days)** | | | | | | | | | | | | |
| 1^44^ | non-randomised studies | not serious | serious^a^ | not serious | not serious | none | 17/345 (4.9%) | 6/72 (8.3%) | **OR 0.57** (0.22 to 1.50) | **34 fewer per 1.000** (from 64 fewer to 37 more) | ⨁◯◯◯ Very low | IMPORTANT |
| **Serious adverse events (assessed with: clinical manifestation,** **follow-up 14 days)** | | | | | | | | | | | | |
| 1^44^ | non-randomised studies | not serious | serious^a^ | not serious | not serious | none | 6/345 (1.7%) | 2/72 (2.8%) | **OR 0.62** (0.12 to 3.13) | **10 fewer per 1.000** (from 24 fewer to 54 more) | ⨁◯◯◯ Very low | IMPORTANT |

**CI:** confidence interval; **OR:** odds ratio

#### Explanations

a. diagnostic uncertainty

References

44. Ambroggio, 2016

***Aminopenicillin compared to broad-spectrum antibiotics for CAP in children with a complete immunization schedule***

| **Certainty assessment** | | | | | | | **№ of patients** | | **Effect** | | **Certainty** | **Importance** |
| --- | --- | --- | --- | --- | --- | --- | --- | --- | --- | --- | --- | --- |
| **№ of studies** | **Study design** | **Risk of bias** | **Inconsistency** | **Indirectness** | **Imprecision** | **Other considerations** | **aminopenicillin** | **broad-spectrum antibiotics** | **Relative (95% CI)** | **Absolute (95% CI)** |  |  |
| **Treatment failure (assessed with: Need of hospital admission/re-admission, follow-up 14 days)** | | | | | | | | | | | | |
| 1^51^ | non-randomised studies | serious^a^ | serious^b^ | not serious | not serious | none | 39/1610 (2.4%) | 321/13954 (2.3%) | **OR 1.05** (0.75 to 1.48) | **1 more per 1.000** (from 6 fewer to 11 more) | ⨁◯◯◯ Very low | IMPORTANT |
| **Treatment failure (assessed with: Progression to severe/complicated pneumonia or admission to ICU, follow-up 14 days)** | | | | | | | | | | | | |
| 1^51^ | non-randomised studies | not serious | serious^b^ | not serious | not serious | none | 13/1610 (0.8%) | 156/13954 (1.1%) | **OR 0.72** (0.41 to 1.27) | **3 fewer per 1.000** (from 7 fewer to 3 more) | ⨁◯◯◯ Very low | IMPORTANT |
| **Treatment failure (assessed with: median length of fever, follow-up 14 days)** | | | | | | | | | | | | |
| 1^49^ | non-randomised studies | serious^a^ | serious^b^ | not serious | not serious | none | 6.5 | 9.1 | - | **0**  (0 to 0 ) | ⨁◯◯◯ Very low | IMPORTANT |
| **Treatment failure (assessed with: Need of hospital admission/re-admission, follow-up 30 days)** | | | | | | | | | | | | |
| 2^48,53^ | non-randomised studies | serious^a^ | serious^b^ | not serious | not serious | none | 2549/20696 (12.3%) | 6494/51648 (12.6%) | **OR 0.98** (0.93 to 1.03) | **3 fewer per 1.000** (from 8 fewer to 3 more) | ⨁◯◯◯ Very low | IMPORTANT |
| **Treatment failure (assessed with: Progression to severe/complicated pneumonia or admission to PICU, follow-up 14 days)** | | | | | | | | | | | | |
| 1^48^ | non-randomised studies | not serious | not serious | not serious | not serious | none | 54/20485 (0.3%) | 248/51319 (0.5%) | **OR 0.54** (0.41 to 0.73) | **2 fewer per 1.000** (from 3 fewer to 1 fewer) | ⨁⨁◯◯ Low | IMPORTANT |

**CI:** confidence interval; **OR:** odds ratio

#### Explanations

a. moderate quality of the study

b. diagnostic uncertainty

References

51. Williams, 2013

49. Queen, 2014

53, Puz, 2023

48, Ambroggio, 2023

**QUESTION 2 - What is the first-line antibiotic for treating mild-moderate CAP in a child over five years old with a complete immunization schedule (at least two doses of hexavalent and pneumococcal vaccines)?**

***Amoxicillin compared to broad-spectrum antibiotics for CAP in children with a complete immunization schedule***

| **Certainty assessment** | | | | | | | **№ of patients** | | **Effect** | | **Certainty** | **Importance** |
| --- | --- | --- | --- | --- | --- | --- | --- | --- | --- | --- | --- | --- |
| **№ of studies** | **Study design** | **Risk of bias** | **Inconsistency** | **Indirectness** | **Imprecision** | **Other considerations** | **Amoxicillina** | **broad-spectrum antibiotics** | **Relative (95% CI)** | **Absolute (95% CI)** |  |  |
| **Treatment failure (assessed with: New antibiotic prescription, follow-up 14 days)** | | | | | | | | | | | | |
| 1^46^ | non-randomised studies | serious^a^ | serious^b^ | not serious | not serious | none | 2909/65872 (4.4%) | 3204/62381 (5.1%) | **OR 0.85**  (0.81 to 0.90) | **7 fewer per 1.000** (from 9 fewer to 5 fewer) | ⨁◯◯◯ Very low | IMPORTANT |
| **Treatment failure (assessed with: need of hospitalization/re-hospitalization, follow-up 14 days)** | | | | | | | | | | | | |
| 1^46^ | non-randomised studies | serious^a^ | serious^b^ | not serious | not serious | none | 428/65872 (0.6%) | 526/62381 (0.8%) | **OR 0.77** (0.68 to 0.87) | **2 fewer per 1.000** (from 3 fewer to 1 fewer) | ⨁◯◯◯ Very low | IMPORTANT |
| **Treatment outcome (assessed with: progression to severe or complicated CAP or admission to ICU, follow-up 14 days)** | | | | | | | | | | | | |
| 1^46^ | non-randomised studies | serious^a^ | serious^b^ | not serious | not serious | none | 35/65872 (0.1%) | 40/62381 (0.1%) | **OR 0.83** (0.53 to 1.30) | **0 fewer per 1.000** (from 0 fewer to 0 fewer) | ⨁◯◯◯ Very low | IMPORTANT |

**CI:** confidence interval; **OR:** odds ratio

#### Explanations

a. moderate quality of study

b. diagnostic uncertainty

References

46. Lipsett, 2021

***Amoxicillin compared to Macrolides for CAP in children with a complete immunization schedule***

| **Certainty assessment** | | | | | | | **№ of patients** | | **Effect** | | **Certainty** | **Importance** |
| --- | --- | --- | --- | --- | --- | --- | --- | --- | --- | --- | --- | --- |
| **№ of studies** | **Study design** | **Risk of bias** | **Inconsistency** | **Indirectness** | **Imprecision** | **Other considerations** | **Amoxicillin** | **Macrolides** | **Relative (95% CI)** | **Absolute (95% CI)** |  |  |
| **Treatment failure (assessed with: New antibiotic prescription, follow-up 14 days)** | | | | | | | | | | | | |
| 1^46^ | non-randomised studies | serious^a^ | serious^b^ | not serious | not serious | none | 2909/65872 (4.4%) | 4697/108917 (4.3%) | **OR 1.03** (0.98 to 1.07) | **1 more per 1.000** (from 1 fewer to 3 more) | ⨁◯◯◯ Very low | IMPORTANT |
| **Treatment failure (assessed with: Need of hospitalization/re-hospitalization, follow-up 14 days)** | | | | | | | | | | | | |
| 1^46^ | non-randomised studies | serious^a^ | serious^b^ | not serious | not serious | none | 428/65872 (0.6%) | 402/108917 (0.4%) | **OR 1.77** (1.54 to 2.02) | **3 more per 1.000** (from 2 more to 4 more) | ⨁◯◯◯ Very low | IMPORTANT |
| **Treatment failure (assessed with: Progression to severe/complicated CAP or admission to ICU, follow-up 14 days)** | | | | | | | | | | | | |
| 1^46^ | non-randomised studies | serious^a^ | serious^b^ | not serious | not serious | none | 35/65872 (0.1%) | 28/108917 (0.0%) | **OR 2.07** (1.26 to 3.40) | **0 fewer per 1.000** (from 0 fewer to 1 more) | ⨁◯◯◯ Very low | IMPORTANT |

**CI:** confidence interval; **OR:** odds ratio

#### Explanations

a. moderate quality of study

b. diagnostic uncertainty

References

46. Lipsett, 2021

***Beta-lactams compared to Macrolides for CAP in children with a complete immunization schedule***

| **Certainty assessment** | | | | | | | **№ of patients** | | **Effect** | | **Certainty** | **Importance** |
| --- | --- | --- | --- | --- | --- | --- | --- | --- | --- | --- | --- | --- |
| **№ of studies** | **Study design** | **Risk of bias** | **Inconsistency** | **Indirectness** | **Imprecision** | **Other considerations** | **betalactams** | **Macrolides** | **Relative (95% CI)** | **Absolute (95% CI)** |  |  |
| **Treatment failure (assessed with: clinical cure, follow-up 7 days)** | | | | | | | | | | | | |
| 5^a,29^ | randomised trials | serious^b^ | not serious | not serious | not serious | publication bias strongly suspected^c^ | 178/255 (69.8%) | 164/190 (86.3%) | **OR 0.37** (0.22 to 0.60) | **163 fewer per 1.000** (from 282 fewer to 72 fewer) | ⨁⨁◯◯ Low | IMPORTANT |

**CI:** confidence interval; **OR:** odds ratio

#### Explanations

a. 5 RCTs included in a meta-analysis

b. most of the studies included in the systematic review have bias (performance, detection, attrition) or conflict of interest

c. publication bias in small studies regarding efficacy of treatment

References

29. Biondi, 2014

***Beta-lactams compared to beta-lactams + Macrolides for CAP in children with a complete immunization schedule***

| **Certainty assessment** | | | | | | | **№ of patients** | | **Effect** | | **Certainty** | **Importance** |
| --- | --- | --- | --- | --- | --- | --- | --- | --- | --- | --- | --- | --- |
| **№ of studies** | **Study design** | **Risk of bias** | **Inconsistency** | **Indirectness** | **Imprecision** | **Other considerations** | **betalactams** | **betalactams + macrolides** | **Relative (95% CI)** | **Absolute (95% CI)** |  |  |
| **Treatment failure (assessed with: new prescription of antibiotic, follow-up 14 days)** | | | | | | | | | | | | |
| 1^46^ | non-randomised studies | serious^a^ | serious^b^ | not serious | not serious | none | 6113/128253 (4.8%) | 273/11719 (2.3%) | **OR 2.10** (1.86 to 2.37) | **24 more per 1.000** (from 19 more to 30 more) | ⨁◯◯◯ Very low | IMPORTANT |
| **Treatment failure (assessed with: Need of hospital admission/re-hospital admission, follow-up 14 days)** | | | | | | | | | | | | |
| 1^46^ | non-randomised studies | serious^a^ | serious^b^ | not serious | not serious | none | 954/128253 (0.7%) | 87/11719 (0.7%) | **OR 1.00** (0.80 to 1.25) | **0 fewer per 1.000** (from 1 fewer to 2 more) | ⨁◯◯◯ Very low | IMPORTANT |
| **Treatment failure (assessed with: Progression to severe/complicated pneumonia or admission to PICU, follow-up 14 days)** | | | | | | | | | | | | |
| 1^46^ | non-randomised studies | serious^a^ | serious^b^ | not serious | not serious | none | 75/128253 (0.1%) | 7/11719 (0.1%) | **OR 0.98** (0.45 to 2.12) | **0 fewer per 1.000** (from 0 fewer to 1 more) | ⨁◯◯◯ Very low | IMPORTANT |
| **Treatment failure (assessed with: Need of hospital admission/re-admission, follow-up 3-10 weeks)** | | | | | | | | | | | | |
| 1^52^ | non-randomised studies | serious^a^ | not serious | not serious | not serious | none | 33/616 (5.4%) | 5/257 (1.9%) | **OR 2.85** (1.10 to 7.39) | **34 more per 1.000** (from 2 more to 108 more) | ⨁◯◯◯ Very low | IMPORTANT |
| **Treatment failure (assessed with: Progression to severe/complicated pneumonia or admission to PICU, follow-up 3-10 weeks)** | | | | | | | | | | | | |
| 1^52^ | non-randomised studies | serious^a^ | not serious | not serious | not serious | none | 57/1017 (5.6%) | 25/399 (6.3%) | **OR 0.89** (0.55 to 1.44) | **7 fewer per 1.000** (from 27 fewer to 25 more) | ⨁◯◯◯ Very low | IMPORTANT |

**CI:** confidence interval; **OR:** odds ratio

#### Explanations

a. moderate quality of study

b. diagnostic uncertainty

References

46. Lipsett, 2021

52. Williams, 2017

***Amoxicillin compared to Macrolides for CAP in children younger than 5 years of age with a complete immunization schedule***

| **Certainty assessment** | | | | | | | **№ of patients** | | **Effect** | | **Certainty** | **Importance** |
| --- | --- | --- | --- | --- | --- | --- | --- | --- | --- | --- | --- | --- |
| **№ of studies** | **Study design** | **Risk of bias** | **Inconsistency** | **Indirectness** | **Imprecision** | **Other considerations** | **Amoxicillin** | **Macrolides** | **Relative (95% CI)** | **Absolute (95% CI)** |  |  |
| **Treatment failure (assessed with: new antibiotic prescription, follow-up 14 days)** | | | | | | | | | | | | |
| 1^42^ | non-randomised studies | not serious | serious^a^ | not serious | not serious | none | 23/243 (9.5%) | 12/243 (4.9%) | **OR 2.01** (0.98 to 4.14) | **45 more per 1.000** (from 1 fewer to 128 more) | ⨁◯◯◯ Very low | IMPORTANT |

**CI:** confidence interval; **OR:** odds ratio

#### Explanations

a. diagnostic uncertainty

References

42. Ambroggio, 2015

***Beta-lactams compared to beta-lactams + Macrolides for CAP in children younger than 5 years of age with a complete immunization schedule***

| **Certainty assessment** | | | | | | | **№ of patients** | | **Effect** | | **Certainty** | **Importance** |
| --- | --- | --- | --- | --- | --- | --- | --- | --- | --- | --- | --- | --- |
| **№ of studies** | **Study design** | **Risk of bias** | **Inconsistency** | **Indirectness** | **Imprecision** | **Other considerations** | **betalactams** | **betalactams + macrolides** | **Relative (95% CI)** | **Absolute (95% CI)** |  |  |
| **Treatment failure (assessed with: New antibiotic prescription, follow-up 14 days)** | | | | | | | | | | | | |
| 1^44^ | non-randomised studies | not serious | serious^a^ | not serious | not serious | none | 29/225 (12.9%) | 3/75 (4.0%) | **OR 3.55** (1.05 to 12.02) | **89 more per 1.000** (from 2 more to 294 more) | ⨁◯◯◯ Very low | IMPORTANT |
| **Serious adverse events (assessed with: Clinical evaluation, follow-up 14 days)** | | | | | | | | | | | | |
| 1^44^ | non-randomised studies | not serious | serious^a^ | not serious | not serious | none | 5/225 (2.2%) | 1/75 (1.3%) | **OR 1.68** (0.19 to 14.63) | **9 more per 1.000** (from 11 fewer to 152 more) | ⨁◯◯◯ Very low |  |

**CI:** confidence interval; **OR:** odds ratio

#### Explanations

a. diagnostic uncertainty

References

44. Ambroggio, 2016

***Aminopenicillin compared to broad-spectrum antibiotics for CAP in children with a complete immunization schedule***

| **Certainty assessment** | | | | | | | **№ of patients** | | **Effect** | | **Certainty** | **Importance** |
| --- | --- | --- | --- | --- | --- | --- | --- | --- | --- | --- | --- | --- |
| **№ of studies** | **Study design** | **Risk of bias** | **Inconsistency** | **Indirectness** | **Imprecision** | **Other considerations** | **aminopenicillin** | **broad-spectrum antibiotics** | **Relative (95% CI)** | **Absolute (95% CI)** |  |  |
| **Treatment failure (assessed with: Need of hospital admission/re-admission, follow-up 14 days)** | | | | | | | | | | | | |
| 1^51^ | non-randomised studies | serious^a^ | serious^b^ | not serious | not serious | none | 39/1610 (2.4%) | 321/13954 (2.3%) | **OR 1.05** (0.75 to 1.48) | **1 more per 1.000** (from 6 fewer to 11 more) | ⨁◯◯◯ Very low | IMPORTANT |
| **Treatment failure (assessed with: Progression to severe/complicated pneumonia or admission to ICU, follow-up 14 days)** | | | | | | | | | | | | |
| 1^51^ | non-randomised studies | not serious | serious^b^ | not serious | not serious | none | 13/1610 (0.8%) | 156/13954 (1.1%) | **OR 0.72** (0.41 to 1.27) | **3 fewer per 1.000** (from 7 fewer to 3 more) | ⨁◯◯◯ Very low | IMPORTANT |
| **Treatment failure (assessed with: median length of fever, follow-up 14 days)** | | | | | | | | | | | | |
| 1^49^ | non-randomised studies | serious^a^ | serious^b^ | not serious | not serious | none | 6.5 | 9.1 | - | **0**  (0 to 0 ) | ⨁◯◯◯ Very low | IMPORTANT |
| **Treatment failure (assessed with: Need of hospital admission/re-admission, follow-up 30 days)** | | | | | | | | | | | | |
| 2^48,53^ | non-randomised studies | serious^a^ | serious^b^ | not serious | not serious | none | 2549/20696 (12.3%) | 6494/51648 (12.6%) | **OR 0.98** (0.93 to 1.03) | **3 fewer per 1.000** (from 8 fewer to 3 more) | ⨁◯◯◯ Very low | IMPORTANT |
| **Treatment failure (assessed with: Progression to severe/complicated pneumonia or admission to PICU, follow-up 14 days)** | | | | | | | | | | | | |
| 1^48^ | non-randomised studies | not serious | not serious | not serious | not serious | none | 54/20485 (0.3%) | 248/51319 (0.5%) | **OR 0.54** (0.41 to 0.73) | **2 fewer per 1.000** (from 3 fewer to 1 fewer) | ⨁⨁◯◯ Low | IMPORTANT |

**CI:** confidence interval; **OR:** odds ratio

#### Explanations

a. moderate quality of the study

b. diagnostic uncertainty

References

51. Williams, 2013

49. Queen, 2014

53, Puz, 2023

48, Ambroggio, 2023

**QUESTION 3 - What is the first line antibiotic for treating mild-moderate CAP in a child without a complete immunization schedule (<2 doses of hexavalent and pneumococcal vaccines)?**

***Aminopenicillin compared to broad-spectrum antibiotics for CAP in children with an incomplete immunization schedule***

| **Certainty assessment** | | | | | | | **№ of patients** | | **Effect** | | **Certainty** | **Importance** |
| --- | --- | --- | --- | --- | --- | --- | --- | --- | --- | --- | --- | --- |
| **№ of studies** | **Study design** | **Risk of bias** | **Inconsistency** | **Indirectness** | **Imprecision** | **Other considerations** | **aminopenicillin** | **broad-spectrum antibiotics** | **Relative (95% CI)** | **Absolute (95% CI)** |  |  |
| **Treatment failure (assessed with: need of new antibiotic prescription,** **follow-up 14 days)** | | | | | | | | | | | | |
| 1^454^ | non-randomised studies | serious^a^ | serious^b^ | not serious | not serious | none | 5/66 (7.6%) | 12/253 (4.7%) | **OR 1.65** (0.56 to 4.85) | **28 more per 1.000** (from 20 fewer to 147 more) | ⨁◯◯◯ Very low | IMPORTANT |
| **Treatment failure (assessed with: fever,** **follow-up 48-72 hours)** | | | | | | | | | | | | |
| 1^54^ | non-randomised studies | serious^a^ | serious^b^ | not serious | not serious | none | 3/66 (4.5%) | 15/253 (5.9%) | **OR 0.76** (0.21 to 2.69) | **14 fewer per 1.000** (from 46 fewer to 86 more) | ⨁◯◯◯ Very low | IMPORTANT |

**CI:** confidence interval; **OR:** odds ratio

#### Explanations

a. moderate quality of study

b. diagnostic uncertainty

References

54. Dinur-Scheijetr, 2013

***Benzylpenicillin (IV) compared to amoxicillin (oral for CAP in children with an incomplete immunization schedule***

| **Certainty assessment** | | | | | | | **№ of patients** | | **Effect** | | **Certainty** | **Importance** |
| --- | --- | --- | --- | --- | --- | --- | --- | --- | --- | --- | --- | --- |
| **№ of studies** | **Study design** | **Risk of bias** | **Inconsistency** | **Indirectness** | **Imprecision** | **Other considerations** | **benzilpenicillin (IV route)** | **amoxicillin (oral route)** | **Relative (95% CI)** | **Absolute (95% CI)** |  |  |
| **Treatment failure (assessed with: Need of new antibiotic prescription,** **follow-up 14 days)** | | | | | | | | | | | | |
| 1^63^ | randomised trials | serious^a^ | not serious | not serious | not serious | none | 2/103 (1.9%) | 6/100 (6.0%) | **OR 3.22** (0.63 to 16.37) | **125 more per 1.000** (from 21 fewer to 451 more) | ⨁⨁⨁◯ Moderate | IMPORTANT |
| **Treatment failure (assessed with: need of hospital/re-hospital admission,** **follow-up 14 days)** | | | | | | | | | | | | |
| 1^63^ | randomised trials | serious^a^ | not serious | not serious | not serious | none | 1/103 (1.0%) | 0/100 (0.0%) | not estimable |  | ⨁⨁⨁◯ Moderate | IMPORTANT |

**CI:** confidence interval; **OR:** odds ratio

#### Explanations

a. moderate quality of study

b. diagnostic uncertainty

References

63. Atkinson, 2007 (Lodha)

**QUESTION 4 - What is the first-line antibiotic in the treatment of mild-moderate bacterial CAP in patients allergic to penicillin?**

***Macrolides compared to beta-lactams for CAP in children with penicillin allergy***

| **Certainty assessment** | | | | | | | **№ of patients** | | **Effect** | | **Certainty** | **Importance** |
| --- | --- | --- | --- | --- | --- | --- | --- | --- | --- | --- | --- | --- |
| **№ of studies** | **Study design** | **Risk of bias** | **Inconsistency** | **Indirectness** | **Imprecision** | **Other considerations** | **Macrolides** | **beta-lactams** | **Relative (95% CI)** | **Absolute (95% CI)** |  |  |
| **Treatment failure (assessed with: New antibiotic prescription, follow-up 14 days)** | | | | | | | | | | | | |
| 2^46,42^ | non-randomised studies | serious^a^ | serious^b^ | not serious | not serious | none | 4718/109289 (4.3%) | 2942/66454 (4.4%) | **OR 0.97** (0.93 to 1.02) | **1 fewer per 1.000** (from 3 fewer to 1 more) | ⨁◯◯◯ Very low | IMPORTANT |
| **Treatment failure (assessed with: Need of hospital/re-hospital admission, follow-up 14 days)** | | | | | | | | | | | | |
| 1^46^ | non-randomised studies | serious^a^ | serious^b^ | not serious | not serious | none | 402/108917 (0.4%) | 2942/66454 (4.4%) | **OR 0.57** (0.50 to 0.65) | **19 fewer per 1.000** (from 22 fewer to 15 fewer) | ⨁◯◯◯ Very low | IMPORTANT |
| **Treatment failure (assessed with: Progression to severe pneumonia or PICU admission, follow-up 14 days)** | | | | | | | | | | | | |
| 1^46^ | non-randomised studies | serious^a^ | serious^b^ | not serious | not serious | none | 28/108917 (0.0%) | 35/65872 (0.1%) | **OR 0.48** (0.29 to 0.80) | **0 fewer per 1.000** (from 0 fewer to 0 fewer) | ⨁◯◯◯ Very low | IMPORTANT |

**CI:** confidence interval; **OR:** odds ratio

#### Explanations

a. moderate quality of study

b. diagnostic uncertainty

References

46. Lipset, 2021

42. Ambroggio, 2015

***Beta-lactams or Macrolides compared to levofloxacin for CAP in children with penicillin allergy***

| **Certainty assessment** | | | | | | | **№ of patients** | | **Effect** | | **Certainty** | **Importance** |
| --- | --- | --- | --- | --- | --- | --- | --- | --- | --- | --- | --- | --- |
| **№ of studies** | **Study design** | **Risk of bias** | **Inconsistency** | **Indirectness** | **Imprecision** | **Other considerations** | **betalactams or macrolides** | **levofloxacin** | **Relative (95% CI)** | **Absolute (95% CI)** |  |  |
| **Treatment failure (assessed with: clinical cure, follow-up 14 days )** | | | | | | | | | | | | |
| 1^61^ | randomised trials | very serious^a^ | not serious | not serious | not serious | none | 126/134 (94.0%) | 382/405 (94.3%) | **OR 0.95** (0.41 to 2.17) | **3 fewer per 1.000** (from 71 fewer to 30 more) | ⨁⨁◯◯ Low | IMPORTANT |

**CI:** confidence interval; **OR:** odds ratio

#### Explanations

a. high risk of performance and detection bias, not estimable risks for other bias

References

61. Bradley, 2007 (Lodha)

***Azithromycin compared to Erythromycin for CAP in children with penicillin allergy***

| **Certainty assessment** | | | | | | | **№ of patients** | | **Effect** | | **Certainty** | **Importance** |
| --- | --- | --- | --- | --- | --- | --- | --- | --- | --- | --- | --- | --- |
| **№ of studies** | **Study design** | **Risk of bias** | **Inconsistency** | **Indirectness** | **Imprecision** | **Other considerations** | **Azithromycin** | **Erythromycin** | **Relative (95% CI)** | **Absolute (95% CI)** |  |  |
| **Treatment failure (assessed with: Progression to severe/complicated pneumonia, follow-up 14 days)** | | | | | | | | | | | | |
| 1^64^ | randomised trials | serious^a^ | not serious | not serious | not serious | none | 1/45 (2.2%) | 4/40 (10.0%) | **OR 0.20** (0.03 to 1.91) | **78 fewer per 1.000** (from 98 fewer to 75 more) | ⨁⨁⨁◯ Moderate | IMPORTANT |
| **Treatment failure (assessed with: New episode of CAP, follow-up 30 days)** | | | | | | | | | | | | |
| 1^64^ | randomised trials | serious^a^ | not serious | not serious | not serious | none | 2/45 (4.4%) | 0/40 (0.0%) | not estimable |  | ⨁⨁⨁◯ Moderate | IMPORTANT |

**CI:** confidence interval; **OR:** odds ratio

#### Explanations

a. high risk of bias for detection and performance bias due to absence of blinding but low risk of bias in reporting bias and attrition bias

References

64. Roord, 1996 (Lodha)

***Clarithromycin compared to Erythromycin for CAP in children with penicillin allergy***

| **Certainty assessment** | | | | | | | **№ of patients** | | **Effect** | | **Certainty** | **Importance** |
| --- | --- | --- | --- | --- | --- | --- | --- | --- | --- | --- | --- | --- |
| **№ of studies** | **Study design** | **Risk of bias** | **Inconsistency** | **Indirectness** | **Imprecision** | **Other considerations** | **Clarithromycin** | **Erythromycin** | **Relative (95% CI)** | **Absolute (95% CI)** |  |  |
| **Treatment (assessed with: Progression to severe/complicated pneumonia or PICU admission**, **follow-up 14 days)** | | | | | | | | | | | | |
| 1^65^ | randomised trials | serious^a^ | not serious | not serious | not serious | none | 3/124 (2.4%) | 3/110 (2.7%) | **OR 0.88** (0.17 to 4.47) | **3 fewer per 1.000** (from 23 fewer to 48 more) | ⨁⨁⨁◯ Moderate | IMPORTANT |
| **Treatment failure (assessed with: New episode of pneumoniae,** **follow-up 30 days)** | | | | | | | | | | | | |
| 1^65^ | randomised trials | serious^a^ | not serious | not serious | not serious | none | 1/121 (0.8%) | 5/105 (4.8%) | **OR 0.17** (0.02 to 1.45) | **39 fewer per 1.000** (from 47 fewer to 20 more) | ⨁⨁⨁◯ Moderate | IMPORTANT |

**CI:** confidence interval; **OR:** odds ratio

#### Explanations

a. high risk of performance bias due to absence of blinding in the participants but low risk of bias in detection bias due to blinding of outcome assessors; low risk or unclear risk of bias in the other domains

References

65. Block, 1995 (Lodha)

**QUESTION 5 - What should be the optimal dosage of amoxicillin in treating mild to moderate bacterial CAP?**

***Amoxicillin 90 mg/kg/day in 2 doses compared to amoxicillin 50 mg/kg/day in 2 doses for CAP in children***

| **Certainty assessment** | | | | | | | **№ of patients** | | **Effect** | | **Certainty** | **Importance** |
| --- | --- | --- | --- | --- | --- | --- | --- | --- | --- | --- | --- | --- |
| **№ of studies** | **Study design** | **Risk of bias** | **Inconsistency** | **Indirectness** | **Imprecision** | **Other considerations** | **Amoxicillin 90 mg/kg/day in 2 doses** | **amoxicillin 50 mg/kg/day in 2 doses** | **Relative (95% CI)** | **Absolute (95% CI)** |  |  |
| **Treatment failure (assessed with: New antibiotic prescription, follow-up 14 days ))** | | | | | | | | | | | | |
| 1^38^ | randomised trials | not serious | serious^a^ | not serious | not serious | none | 25/404 (6.2%) | 29/410 (7.1%) | **OR 0.87** (0.50 to 1.47) | **9 fewer per 1.000** (from 34 fewer to 32 more) | ⨁⨁⨁◯ Moderate | IMPORTANT |
| **Treatment failure (assessed with: New prescription of antibiotic, follow-up 30days)** | | | | | | | | | | | | |
| 1^38^ | randomised trials | not serious | serious^a^ | not serious | not serious | none | 49/404 (12.1%) | 51/410 (12.4%) | **OR 0.97** (0.64 to 1.48) | **3 fewer per 1.000** (from 41 fewer to 49 more) | ⨁⨁⨁◯ Moderate | IMPORTANT |
| **Serious adverse events (assessed with: clinical presentation, follow-up 14 days)** | | | | | | | | | | | | |
| 1^38^ | randomised trials | not serious | serious^a^ | not serious | not serious | none | 20/404 (5.0%) | 23/404 (5.7%) | **OR 0.86** (0.47 to 1.60) | **8 fewer per 1.000** (from 29 fewer to 31 more) | ⨁⨁⨁◯ Moderate | IMPORTANT |
| **Antibiotic resistance to penicillin (assessed with: multidrug resistance bacteria, follow-up 30 days)** | | | | | | | | | | | | |
| 1^38^ | randomised trials | not serious | serious^a^ | not serious | not serious | none | 9/213 (4.2%) | 12/224 (5.4%) | **RR 0.78** (0.32 to 1.89) | **11 fewer per 1.000** (from 36 fewer to 43 more) | ⨁⨁⨁◯ Moderate | IMPORTANT |

**CI:** confidence interval; **OR:** odds ratio

#### Explanations

a. diagnostic uncertainty

References

38. Bielicki et al.

**QUESTION 6 - What should be the optimal length of therapy with amoxicillin for treating mild -moderate bacterial CAP?**

***10 days of amoxicillin compared to 7 days of amoxicillin for CAP in children***

| **Certainty assessment** | | | | | | | **№ of patients** | | **Effect** | | **Certainty** | **Importance** |
| --- | --- | --- | --- | --- | --- | --- | --- | --- | --- | --- | --- | --- |
| **№ of studies** | **Study design** | **Risk of bias** | **Inconsistency** | **Indirectness** | **Imprecision** | **Other considerations** | **10 days of amoxicillin** | **7 days of amoxicillin** | **Relative (95% CI)** | **Absolute (95% CI)** |  |  |
| **Treatment failure (assessed with: new antibiotic prescription, follow-up 14 days)** | | | | | | | | | | | | |
| 1^47,c^ | non-randomised studies | serious^a^ | serious^b^ | not serious | not serious | none | 6848/109035 (6.3%) | 729/12811 (5.7%) | **OR 1.11** (1.03 to 1.20) | **6 more per 1.000** (from 1 more to 11 more) | ⨁◯◯◯ Very low | IMPORTANT |
| **Treatment failure (assessed with: Need of hospital/re-hospital admission, follow-up 14 days)** | | | | | | | | | | | | |
| 1^47,c^ | non-randomised studies | serious^a^ | serious^b^ | not serious | not serious | none | 231/109035 (0.2%) | 35/12811 (0.3%) | **OR 0.77** (0.54 to 1.11) | **1 fewer per 1.000** (from 1 fewer to 0 fewer) | ⨁◯◯◯ Very low | IMPORTANT |

**CI:** confidence interval; **OR:** odds ratio

#### Explanations

a. moderate quality of study

b. diagnostic uncertainty

c. short therapy (5-9 days) compared to long therapy (10-14 days)

References

47. Saphiro, 2021

***10 days of amoxicillin compared to 5 days of amoxicillin for CAP in children***

| **Certainty assessment** | | | | | | | **№ of patients** | | **Effect** | | **Certainty** | **Importance** |
| --- | --- | --- | --- | --- | --- | --- | --- | --- | --- | --- | --- | --- |
| **№ of studies** | **Study design** | **Risk of bias** | **Inconsistency** | **Indirectness** | **Imprecision** | **Other considerations** | **10 days of amoxicillin** | **5 days of amoxicillin** | **Relative (95% CI)** | **Absolute (95% CI)** |  |  |
| **Treatment failure (assessed with: New antibiotic prescription, follow-up 14 days)** | | | | | | | | | | | | |
| 1^40^ | randomised trials | not serious | serious^a^ | not serious | not serious | none | 1/191 (0.5%) | 2/189 (1.1%) | **OR 0.49** (0.04 to 5.47) | **5 fewer per 1.000** (from 10 fewer to 45 more) | ⨁⨁⨁◯ Moderate | IMPORTANT |
| **Treatment failure (assessed with: Need of hospital/re-hospital admission, follow-up 14 days)** | | | | | | | | | | | | |
| 1^40^ | randomised trials | not serious | serious^a^ | not serious | not serious | none | 0/191 (0.0%) | 0/189 (0.0%) | not estimable |  | ⨁⨁⨁◯ Moderate | IMPORTANT |
| **Treatment failure (assessed with: Need of hospital/re-hospital admission, follow-up 30 days)** | | | | | | | | | | | | |
| 2^39,41^ | randomised trials | serious^b^ | serious^a^ | not serious | not serious | none | 12/184 (6.5%) | 8/186 (4.3%) | **OR 1.55** (0.62 to 3.89) | **22 more per 1.000** (from 16 fewer to 106 more) | ⨁⨁◯◯ Low | IMPORTANT |
| **Treatment failure (assessed with: New antibiotic prescription for new episode of CAP, follow-up 30 days)** | | | | | | | | | | | | |
| 1^40^ | randomised trials | not serious | serious^a^ | not serious | not serious | none | 3/191 (1.6%) | 2/189 (1.1%) | **OR 1.49** (0.25 to 9.03) | **5 more per 1.000** (from 8 fewer to 77 more) | ⨁⨁⨁◯ Moderate | IMPORTANT |
| **Treatment failure (assessed with: Fever, follow-up 48-72 hours)** | | | | | | | | | | | | |
| 1^40^ | randomised trials | not serious | serious^a^ | not serious | not serious | none | 1/191 (0.5%) | 2/189 (1.1%) | **OR 0.49** (0.04 to 5.47) | **5 fewer per 1.000** (from 10 fewer to 45 more) | ⨁⨁⨁◯ Moderate | IMPORTANT |
| **Treatment failure (assessed with: cough, follow-up 5 days)** | | | | | | | | | | | | |
| 1^40^ | randomised trials | not serious | serious^a^ | not serious | not serious | none | 6/191 (3.1%) | 7/189 (3.7%) | **OR 0.84** (0.28 to 2.56) | **6 fewer per 1.000** (from 26 fewer to 53 more) | ⨁⨁⨁◯ Moderate | IMPORTANT |
| **Serious Adverse Events (assessed with: Clinical presentation, follow-up 14 days)** | | | | | | | | | | | | |
| 2^40,39^ | randomised trials | serious^b^ | serious^a^ | not serious | not serious | none | 2/332 (0.6%) | 1/329 (0.3%) | **OR 1.99** (0.18 to 22.03) | **3 more per 1.000** (from 2 fewer to 60 more) | ⨁⨁◯◯ Low | IMPORTANT |

**CI:** confidence interval; **OR:** odds ratio

#### Explanations

a. diagnostic uncertainty

b. selective reporting bias in Greenberg's study

References

40. Williams, 2022 (17)

39. Pernica, 2018 (18)

41. Greenberg, 2014 (19)

***7 days of amoxicillin compared to 3 days of amoxicillin for CAP in children***

| **Certainty assessment** | | | | | | | **№ of patients** | | **Effect** | | **Certainty** | **Importance** |
| --- | --- | --- | --- | --- | --- | --- | --- | --- | --- | --- | --- | --- |
| **№ of studies** | **Study design** | **Risk of bias** | **Inconsistency** | **Indirectness** | **Imprecision** | **Other considerations** | **7 days amoxicillin** | **3 days amoxicillin** | **Relative (95% CI)** | **Absolute (95% CI)** |  |  |
| **Treatment failure (assessed with: New antibiotic prescription, follow-up 14 days)** | | | | | | | | | | | | |
| 1^38^ | randomised trials | not serious | serious^a^ | not serious | not serious | none | 26/401 (6.5%) | 28/410 (6.8%) | **OR 0.95** (0.54 to 1.64) | **3 fewer per 1.000** (from 30 fewer to 39 more) | ⨁⨁⨁◯ Moderate | IMPORTANT |
| **Treatment failure (assessed with: New antibiotic prescription for new episode of CAP, follow-up 30 days)** | | | | | | | | | | | | |
| 1^38^ | randomised trials | not serious | serious^a^ | not serious | not serious | none | 49/404 (12.1%) | 51/410 (12.4%) | **OR 0.97** (0.64 to 1.48) | **3 fewer per 1.000** (from 41 fewer to 49 more) | ⨁⨁⨁◯ Moderate | IMPORTANT |
| **Serious Adverse Events (assessed with: Clinical Presentation, follow-up 14 days)** | | | | | | | | | | | | |
| 1^38^ | randomised trials | not serious | serious^a^ | not serious | not serious | none | 18/401 (4.5%) | 25/410 (6.1%) | **OR 0.72** (0.39 to 1.35) | **16 fewer per 1.000** (from 36 fewer to 20 more) | ⨁⨁⨁◯ Moderate | IMPORTANT |
| **Antibiotic resistant to penicillin (assessed with: new antibiotic resistance, follow-up 30 days)** | | | | | | | | | | | | |
| 1^38^ | randomised trials | not serious | serious^a^ | not serious | not serious | none | 7/232 (3.0%) | 14/205 (6.8%) | **OR 0.42** (0.17 to 1.07) | **38 fewer per 1.000** (from 56 fewer to 4 more) | ⨁⨁⨁◯ Moderate | IMPORTANT |

**CI:** confidence interval; **OR:** odds ratio

#### Explanations

a. diagnostic uncertainty

References

38. Bielicki et al.

**QUESTION 7 - What is the most appropriate antibiotic therapy in a child with CAP experiencing clinical deterioration after 48 hours of first-line therapy with amoxicillin?**

***Aminopenicillin compared to broad-spectrum antibiotics for CAP in children with treatment failure after 48 hours of appropriate antibiotic therapy***

| **Certainty assessment** | | | | | | | **№ of patients** | | **Effect** | | **Certainty** | **Importance** |
| --- | --- | --- | --- | --- | --- | --- | --- | --- | --- | --- | --- | --- |
| **№ of studies** | **Study design** | **Risk of bias** | **Inconsistency** | **Indirectness** | **Imprecision** | **Other considerations** | **aminopenicillin** | **broad-spectrum antibiotics** | **Relative (95% CI)** | **Absolute (95% CI)** |  |  |
| **Treatment failure (assessed with: New antibiotic prescription,** **follow-up 14 days)** | | | | | | | | | | | | |
| 1^55^ | non-randomised studies | serious^a^ | serious^b^ | not serious | not serious | none | 15/102 (14.7%) | 23/235 (9.8%) | **OR 1.59** (0.79 to 3.19) | **49 more per 1.000** (from 19 fewer to 159 more) | ⨁◯◯◯ Very low | IMPORTANT |
| **Treatment failure (assessed with: Median duration of fever,** **follow-up 14 days)** | | | | | | | | | | | | |
| 1^55^ | non-randomised studies | serious^a^ | serious^b^ | not serious | not serious | none | 1.8 | 1.2 | - | **0**  (0 to 0 ) | ⨁◯◯◯ Very low | IMPORTANT |

**CI:** confidence interval; **OR:** odds ratio

#### Explanations

a. moderate quality of study

b. diagnostic uncertainty

References

55. Breuer, 2015

1. Boulkedid R, Abdoul H, Loustau M, et al. Using and Reporting the Delphi Method for Selecting Healthcare Quality Indicators: A Systematic Review. PLoS One. 2011;6:e20476 [↑](#endnote-ref-1)
2. Brouwers M, Kho ME, Browman GP, et al. for the AGREE Next Steps Consortium. AGREE II: Advancing guideline development, reporting and evaluation in healthcare. Can Med Assoc J. 2010. Available online July 5, 2010 [↑](#endnote-ref-2)
3. SNLG. Good clinical-care practices. Available in <https://snlg.iss.it/?cat=4> (last accessed 24-07-2021) [↑](#endnote-ref-3)
4. Shea BJ, Reeves BC, Wells G, et al. AMSTAR 2: a critical appraisal tool for systematic reviews that include randomised or non-randomised studies of healthcare interventions, or both. BMJ. 2017; 358:J4008 [↑](#endnote-ref-4)
5. Higgins, J.P.T.; Thomas, J.; Chandler, J.; Cumpston, M.; Li, T.; Page, M.J.; Welch, V.A. (Eds.). Cochrane Handbook for Systematic Reviews of Interventions Version 6.2 (Updated February 2021); Cochrane: 2021. Available online: www.training.cochrane.org/handbook (accessed on 15 July 2022). [↑](#endnote-ref-5)
6. Sterne, J.A.C.; Hernán, M.A.; Reeves, B.C.; Savović, J.; Berkman, N.D.; Viswanathan, M.; Henry, D.; Altman, D.G.; Ansari, M.T.; Boutron, I.; et al. ROBINS-I: A tool for assessing risk of bias in non-randomized studies of interventions. BMJ 2016, 355, i4919e [↑](#endnote-ref-6)
7. Wells GA, Shea B, O'Connell D, et al. The Newcastle-Ottawa Scale (NOS) for assessing the quality of nonrandomized studies in meta-analyses, 2012. Available at: <http://wwwohrica/programs/clinical_epidemiology/oxfordasp> (last accessed 24-07-2021) [↑](#endnote-ref-7)
8. Schünemann HJ, Oxman AD, Brozek J, et al. GRADE Working Group. Grading quality of evidence and strength of recommendations for diagnostic tests and strategies. BMJ. 2008; 336:1106-10 [↑](#endnote-ref-8)
9. Guyatt GH, Oxman AD, Kunz R, et al. GRADE Working Group. Going from evidence to recommendations. BMJ. 2008;336:1049-51 [↑](#endnote-ref-9)
10. Guyatt GH, Oxman AD, Kunz R, et al. GRADE working group. Incorporating considerations of resource use into grading recommendations. BMJ. 2008;336:1170-73 [↑](#endnote-ref-10)
11. [↑](#footnote-ref-1)
