# Supplementary material for: Treatment of mild to moderate community-acquired pneumonia in previously healthy children: an Italian intersociety consensus (SIPPS-SIP-SITIP-FIMP-SIAIP-SIMRI-FIMMG-SIMG)
Source: Ital J Pediatr. 2024 Oct 19;50:217. doi: 10.1186/s13052-024-01786-8 (PMC11491012; doi:10.1186/s13052-024-01786-8)
Supplement: Supplementary file 1 — Supplementary Material 1 [file 13052_2024_1786_MOESM1_ESM.docx]

Padova, September 25^th^, 2024

Manuscript ID: ITJP-D-24-00588

We would like to express our sincere gratitude to both the editor and the reviewers for their thorough evaluation and constructive feedback. We have carefully addressed all comments and suggestions, and a detailed point-by-point response is provided alongside the revised manuscript. Each modification is clearly outlined, and we believe these revisions have substantially improved the quality and clarity of the manuscript.

We have also revised the title to ensure consistency with the other three articles in the Italian intersociety consensus on the judicious use of antibiotic therapy in pediatric respiratory tract infections (pharyngitis, otitis, and sinusitis), which have been submitted to your journal.

We confirm that the data presented in this article are original, unpublished, and not under consideration for publication elsewhere.

All listed authors have made significant contributions to the conception and design of the study, the analysis and interpretation of the data, and the drafting or revision of the manuscript. Each author has reviewed and approved the final version of the manuscript for submission.

We appreciate your consideration of our revised submission and look forward to your feedback.

Thank you again for considering this revised manuscript. I look forward to hearing from you soon.

Best Regards,

Daniele Dona’, MD, PhD on behalf of all co-authors

Corresponding author

Daniele Dona’

Pediatric Infectious Disease Unit, Department for Woman and Child Health, University of Padua, Via Giustiniani 3, 35141, Padua, Italy.

Phone 0039 3388946412

Email: daniele.dona@unipd.it
